# Detection of dynamic protein complexes through Markov Clustering based on Elephant Herd Optimization Approach

**DOI:** 10.1038/s41598-019-47468-y

**Published:** 2019-07-31

**Authors:** R. Ranjani Rani, D. Ramyachitra, A. Brindhadevi

**Affiliations:** Department of Computer Science, Bharathiar University, Tamilnadu, India

**Keywords:** Computational biology and bioinformatics, Data mining

## Abstract

The accessibility of a huge amount of protein-protein interaction (PPI) data has allowed to do research on biological networks that reveal the structure of a protein complex, pathways and its cellular organization. A key demand in computational biology is to recognize the modular structure of such biological networks. The detection of protein complexes from the PPI network, is one of the most challenging and significant problems in the post-genomic era. In Bioinformatics, the frequently employed approach for clustering the networks is Markov Clustering (MCL). Many of the researches for protein complex detection were done on the static PPI network, which suffers from a few drawbacks. To resolve this problem, this paper proposes an approach to detect the dynamic protein complexes through Markov Clustering based on Elephant Herd Optimization Approach (DMCL-EHO). Initially, the proposed method divides the PPI network into a set of dynamic subnetworks under various time points by combining the gene expression data and secondly, it employs the clustering analysis on every subnetwork using the MCL along with Elephant Herd Optimization approach. The experimental analysis was employed on different PPI network datasets and the proposed method surpasses various existing approaches in terms of accuracy measures. This paper identifies the common protein complexes that are expressively enriched in gold-standard datasets and also the pathway annotations of the detected protein complexes using the KEGG database.

## Introduction

The protein complexes are molecular combinations of proteins accumulated by multiple PPI networks, which plays a significant part in numerous biological processes. Several proteins are biologically functional only when they interact with their neighbour proteins and create their protein complex. It is crucial to recognize the sets of proteins that form complexes. Thus, numerous computational approaches have been developed to detect and predict protein complexes from the PPI networks.

High-throughput approaches have created a huge quantity of protein interactions that helps to discover the protein complexes from a large PPI network. During the clustering process, the PPI network is considered as an undirected graph N_et_ = (V_er_, E_dg_) where V_er_ is the set of nodes and E_dg_ is the set of edges. The set of nodes signifies the proteins and set of edges signifies the interaction between proteins.

To cluster the PPI, the network has been modelled into two types, static PPI network that detects the protein functional modules and the second is the dynamic PPI network that detects protein complexes. The dynamic PPI network is defined as the division of static PPI in a series of time-sequenced subnetworks using gene expression data. There exists the variance between protein functional module and protein complexes. The protein functional module is defined as the cluster of proteins which contributes to a specific cellular process and binds with each other at various time points, whereas protein complexes are defined as the cluster of proteins that interacts with each other at the same time point^1^.

Many computational approaches of protein complex detection have been focused on static PPI that extract the dense region in PPI networks, which concentrates only on the topological structure of PPI. Some of the methods that use the static PPI for protein complex detection are MCode^2^, CFinder^3^, MCL^4^, COACH^5^, ClusterOne^6^, RNSC^7^, CMC^8^, and many more. Maulik *et al*., identified the protein complexes using non-cooperative sequential game^9^.

As PPI network continuously transforms with respect to the environment, time and various phases of the cell cycle, the clustering analysis on static PPI does not emulate these dynamic attributes and it is far from optimal solution. Thus, in recent times, various attempts on the clustering process of dynamic PPI network has been initiated along with the gene expression data to enhance the protein complex detection. Also, many evolutionary approaches were employed for analysing the clustering process of the PPI network such as ant colony optimization ACC-DPC^1^, ACO-MCL^10^, cuckoo search optimization (CSO)^11^, BiCAMWI using genetic algorithm^12^, Soft Regularized-MCL^13^, particle swarm optimization (PSO-MCL)^14^ and artificial fish school algorithm (AFA-MCL)^15^. The firefly optimization was employed along with Markov Clustering (F-MCL) on the dynamic PPI network for predicting complexes. The execution time for F-MCL is higher as all the fireflies (proteins) in the population (network) tries to reach the optimal solution (cluster). There are few proteins that are not eligible to come under the cluster and take more iterations to reach the cluster, which may take a long time^16^.

The above-mentioned approaches were effective, but they do not promise a global outcome since they suffer from the effect of unwanted clusters which leads to time consuming. In order to discard the drawbacks of the above-mentioned approaches, a novel approach was proposed to detect the dynamic protein complexes through Markov Clustering based on Elephant Herd Optimization Approach. One of the most important advantages for EHO is that it is the most computationally efficient and has less time consuming compared to F-MCL and other approaches. This is because the unwanted noisy data (unclustered proteins) will be removed from the clan separating operation of EHO approach. The remaining sections of this paper is ordered as follows: Section 2 discusses briefly about the methodology of the proposed approach. Section 3 illustrates the experimental results with various performance measures, Section 4 deliberates about the implementation and discussion of the proposed method in detail and finally Section 5 concludes the paper and recommends for the future enrichments.

## Methods

For detecting the protein complexes, initially, the proposed method divides a static PPI network into a sequence of subnetworks below diverse time points by combining gene expression data to form dynamic model. In order to build a dynamic model, the static PPI network is integrated with gene expression data, which declare the level of gene expression, as well as protein expression. As a protein does not always becomes active at a cell cycle, it is assumed that a protein was active at the time points with its highest expression level^17^. The expression level of a protein will be increased before its expression and will be decreased once the protein has completed its function, and the time points are identified with its expression level, which are higher than a threshold.

Given is a static PPI network *P*_*P*_ = (*P*_*ver*_, *P*_*Edg*_), where *P*_*ver*_, is a set of proteins and *P*_*Edg*_, is a set of interactions between these proteins. In gene expression data, there is a series of *T* time stamps coming with *|P*_*ver*_| × (*T* * *TR*) matrix *M*, where *TR* is the number of repetitions of the time series. Each element *M*(*P*_*ver*_, *j*) of this matrix represents the level of gene expression.

The three-sigma principle is employed to determine if a gene is expressed in a single stamp. For each gene *P*_*ver*_, the gene expression is defined as given in the following Eqs (1–5)

1 $${{\rm{Ev}}}_{{\rm{i}}}({{\rm{P}}}_{{\rm{ver}}})=\frac{{\sum }_{{\rm{tr}}=1}^{{\rm{TR}}}{\rm{M}}({{\rm{P}}}_{{\rm{ver}}},{\rm{i}}+{\rm{T}}\times ({\rm{tr}}-1))}{{\rm{TR}}}$$

2 $${\rm{UE}}({{\rm{P}}}_{{\rm{ver}}})=\frac{{\sum }_{{\rm{i}}=1}^{{\rm{T}}}{{\rm{Ev}}}_{{\rm{i}}}({{\rm{P}}}_{{\rm{ver}}})}{{\rm{T}}}$$

3 $${{\rm{\sigma }}}^{2}({{\rm{P}}}_{{\rm{ver}}})=\frac{{\sum }_{{\rm{i}}=1}^{{\rm{T}}}{({{\rm{Ev}}}_{{\rm{i}}}({{\rm{P}}}_{{\rm{ver}}})-{\rm{UE}}({{\rm{P}}}_{{\rm{ver}}}))}^{2}}{{\rm{T}}}$$

4 $${\rm{Fl}}({{\rm{P}}}_{{\rm{ver}}})=\frac{1}{1+{{\rm{\sigma }}}^{2}({{\rm{P}}}_{{\rm{ver}}})}$$

5 $$\begin{array}{c}{\rm{AT}}({{\rm{P}}}_{{\rm{ver}}})={{\rm{S}}}_{1}({{\rm{P}}}_{{\rm{ver}}})\times {\rm{Fl}}({{\rm{P}}}_{{\rm{ver}}})+{{\rm{S}}}_{2}({{\rm{P}}}_{{\rm{ver}}})\times (1-{\rm{fl}}({{\rm{P}}}_{{\rm{ver}}}))\\ \,\,\,\,\,\,=\,{\rm{UE}}({{\rm{P}}}_{{\rm{ver}}})+3{\rm{\sigma }}({{\rm{P}}}_{{\rm{ver}}})\,(1-{\rm{fl}}({{\rm{P}}}_{{\rm{ver}}}))\end{array}$$

where *Ev*_*i*_(*P*_*ver*_) is the mean of the expression value of gene *P*_*ver*_ at timestamp *i*, *UE*(*P*_*ver*_) is the mean of its expression values over times ranging from 1 to *T*, *σ*(*P*_*ver*_) is the standard deviation of its expression values, *Fl*(*P*_*ver*_) is used to show fluctuation of the expression curve of gene *P*_*ver*_. Suppose that the gene expression data is governed by a normal distribution, then *S*_1_(*P*_*ver*_) and *S*_2_(*P*_*ver*_) are the associated mean and three-sigma value, that is *S*_1_(*P*_*ver*_) = *UE*(*P*_*ver*_) and *S*_2_(*P*_*ver*_) = *UE*(*P*_*ver*_) + 3*σ*(*P*_*ver*_). In virtue of three-sigma principle, the probability that a value greater than *S*_2_(*P*_*ver*_) is not an active point is less than 0.1%. *AT*(*P*_*ver*_) is the active threshold of gene *P*_*ver*_. Consider the gene (*P*_*ver*_) at timestamp *i*. If *Ev*_*i*_(*P*_*ver*_) > *AT*(*P*_*ver*_), then the gene *P*_*ver*_ is expressed and the gene product exists^16^.

In the clustering procedure of every subnetwork, the proposed method starts with constructing the initial protein clusters depending on the protein complexes attained at the prior time point. The initial clusters constructed in the first generation have three steps The procedure for constructing initial clusters has three steps: seed node selection, attachment nodes addition and finally refining^1^. To clearly demonstrate the three steps, a subnetwork of time point *t* with ${P}_{p}^{t}$ = (${P}_{ver}^{t}$, ${P}_{Edg}^{t}$), where ${P}_{ver}^{t}$, is a set of proteins and ${P}_{Edg}^{t}$, is a set of interactions between these proteins at the time *t*.

1. **Selecting seed nodes**: This step first computes the clustering coefficient of every node. Then it selects the nodes whose clustering coefficients are greater than a given threshold *λ*_*c*_ as seed nodes, and puts them into the set of seed nodes at the current time point *t*, denoted by *S*^*t*^. The seed nodes are considered as the candidate clustering centers and represent different clusters of protein complexes. The clustering coefficient of any node *i* is defined in Eq. (6):
  6 $${\rm{\Psi }}=\frac{2\times {n}_{i}^{t}}{|Neigh(i)|\times (|Neigh(i)|-1)}$$
  where *Neigh*(*i*) = {*j є* ${P}_{ver}^{t}$|(*i* · *j*) *є* ${P}_{Edg}^{t}$} represents the neighbor nodes of node *i*, and *|Neigh*(*i*)*|* is the number of neighbor nodes of node *i*, ${n}_{i}^{t}$ is the number of links between neighbour nodes of *i* at the time point *t*.
2. **Attachment nodes addition**: For any seed node *i* (*i* *є* ${S}^{t}$) of current time point *t*, if it is also the seed node of previous time point (*t* − 1), then the nodes which are in the cluster *i* at the previous time point (*t* − 1) and also exists in the subnetwork ${P}_{p}^{t}$ at the current time point *t* are put into the cluster *i* of current time point *t*. In this way, initial clusters are built. However, some clusters may be too sparse since that not all proteins of previous time point (*t* − 1) exist at the current time point *t*. Thus, a refining step is needed to be carried out on the initial clusters.
3. **Refining**: For any initial cluster of protein complex ${c}_{i}^{t}$ at the current time point *t*, if its density is smaller than a given threshold *λ*_*d*_ all the nodes in ${c}_{i}^{t}$ are sorted in a descending order according to their clustering coefficients, and the node with the smallest clustering coefficient is iteratively removed until the density of cluster ${c}_{i}^{t}{\rm{i}}$ is not smaller than the given threshold *λ*_*d*_. The density of a protein complex ${c}_{i}^{t}$ is computed by Eq. (7):

7 $$den({c}_{i}^{t})\frac{2\times {l}_{i}}{{n}_{i}\times ({n}_{i}-1)}$$

where *n*_*i*_ and *l*_*i*_ are number of nodes and edges in cluster ${c}_{i}^{t}$ respectively^1^.

Now, the clustering analysis of the remaining generations is employed by utilizing the Markov Clustering technique along with the EHO algorithm on every subnetwork. The matrix is constructed that depicts the probabilities of transition of a Markov Chain (random walk) based on the graph. The MCL procedure comprises of two activities such as expansion and inflation, which was applied to the matrix that was constructed. The construction of matrix *M*_*at*_ for a graph description and the process of Markov clustering method is briefly described^18^.

Let *P*_*p*_ = (*P*_*ver*_, *P*_*Edg*_), where *P*_*ver*_, is a set of proteins and *P*_*Edg*_, is a set of interactions between these proteins. Denote a node in *P*_*ver*_ by *p*_*vi*_ and an edge between *p*_*vi*_ and *p*_*vj*_ in *P*_*Edg*_ by (*p*_*vi*_, *p*_*vj*_), in which *i* and *j* are the indexes of the corresponding nodes^16^. *W*(*p*_*vi*_, *p*_*vj*_) is the weight of edge (*p*_*vi*_, *p*_*vj*_), which represents the confidence level of the interaction in a weighted PPI networks. *Adj* is the adjacency matrix of a weighted graph given as Eq. (8),

8 $${\rm{Adj}}({\rm{i}},{\rm{j}})=\{\begin{array}{ll}{\rm{W}}({{\rm{p}}}_{{\rm{vi}}},{{\rm{p}}}_{{\rm{vj}}}) & {\rm{if}}\,({{\rm{p}}}_{{\rm{vi}}},{{\rm{p}}}_{{\rm{vj}}})\in {{\rm{P}}}_{{\rm{Edg}}}\\ {{\rm{\max }}}_{{\rm{x}}\ne {\rm{j}}}\,{\rm{W}}({{\rm{p}}}_{{\rm{vi}}},{{\rm{p}}}_{{\rm{vj}}}) & {\rm{if}}\,({{\rm{p}}}_{{\rm{vi}}}={{\rm{p}}}_{{\rm{vj}}})\\ 0 & {\rm{else}}\end{array}$$

A canonical flow matrix *M*_*at*_ is an *n* × *n* (*n* = *|P*_*ver*_*|*) matrix that shows the probabilities of transition of a random walk defined on the graph. *M*_*at*_(*i*, *j*) represents the probability of a transition from node *p*_*vi*_ *to p*_*vj*_. The transition probability from *p*_*vi*_ *to p*_*vj*_ is referred to as the stochastic flow from *p*_*vi*_ *to p*_*vj*_. All the elements in each column of *M*_*at*_ will sum up to 1 and the matrix is expressed as given in Eq. (9)

9 $${{\rm{M}}}_{{\rm{at}}}({\rm{i}},{\rm{j}})=\frac{{\rm{Adj}}({\rm{i}},{\rm{j}})}{{\sum }_{{\rm{k}}=1}^{{\rm{n}}}{\rm{Adj}}({\rm{k}},{\rm{j}})}$$

**Figure 1:**
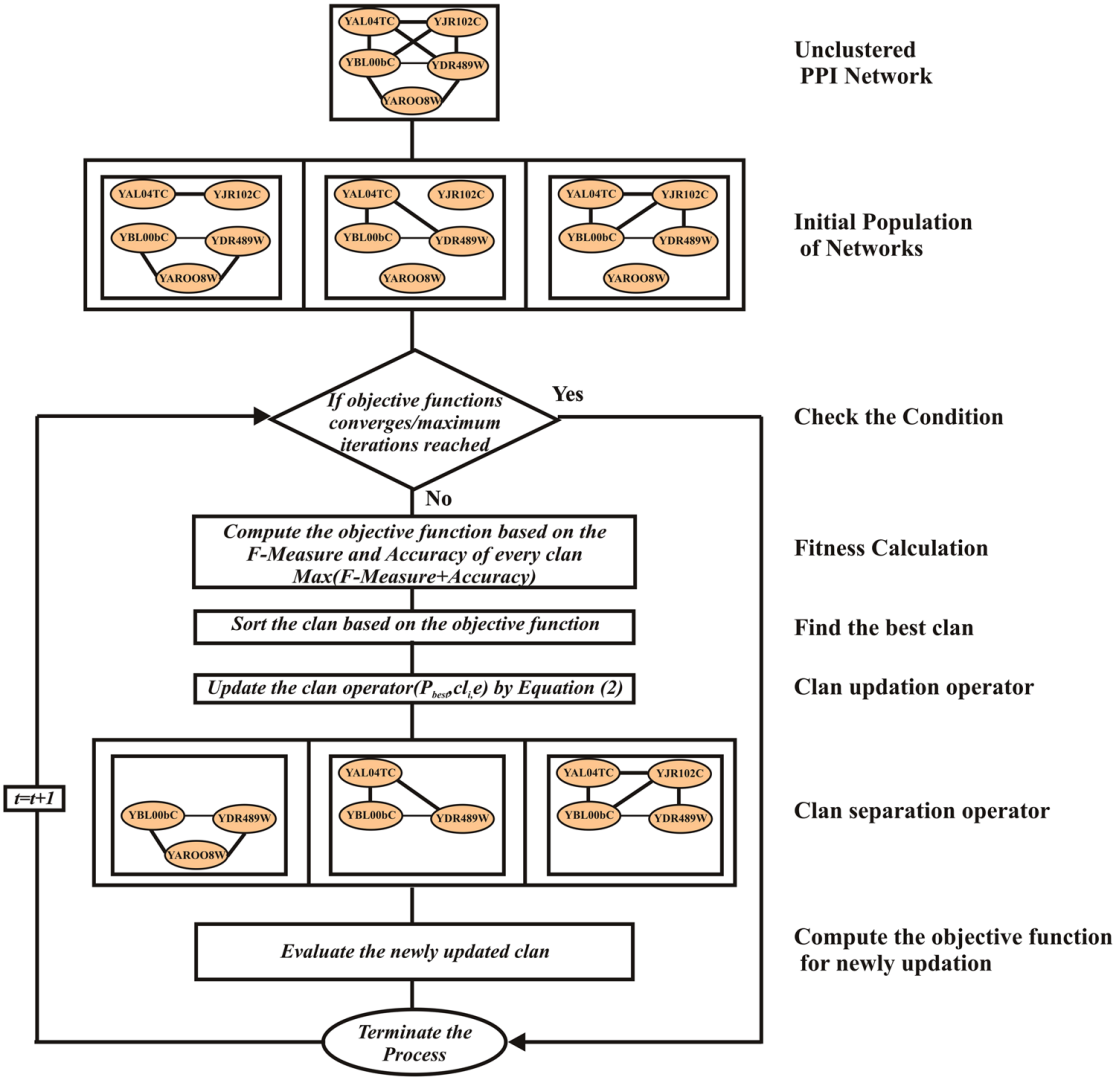
Overall Flowchart of the proposed method.

The three crucial parameters of MCL are inflation constant (*ic*), balance (*b*) and penalty proportion (*P*_*p*_), where *ic* defines the size of each cluster, *b* defines the user-specific balance constant that is employed for penalizing higher-propensity neighbours and *P*_*p*_ defines the penalty ratio of the protein nodes, which is also user-specified^16^. The clustering process using EHO algorithm is briefly explained here for clustering protein complexes. The overall flowchart of the proposed method is shown in Fig. 1.

### Elephant herd optimization

One of the contemporary swarm intelligence technique is the elephant herd optimization which was projected in 2016^19^. This algorithm was stimulated by the herding characteristics of elephants. In general, elephants are social mammals with the composite social group comprising of numerous clans under the guidance of a matriarch. A clan comprises of one or more female elephant with their calves. Female desires to live in domestic clusters while male elephants prefer to live alone and they will exit from the clan when they grow with each generation^20^. The characteristics of the clans signifies exploitation and leaving elephants signifies the exploration of the population.

The characteristics of an elephant are measured using two main operators, namely clan updating and clan separating operators that are used for producing better clustering of proteins. Here, the elephant population is referred to as the static PPI network, each clan is referred to as the dynamic PPI subnetwork, and the elephants inside each clan is represented as proteins.

#### Clan updating operator

The static PPI is initially separated into *k* dynamic PPI. Each dynamic PPI is headed by the individual protein, which represents the best solution of the dynamic PPI. In each generation, protein *e* of dynamic PPI *cl*_*i*_ moves towards the ${p}_{best,c{l}_{i}}$ which has the best fitness in dynamic PPI *cl*_*i*_. The fitness of the dynamic PPI is computed by employing the accuracy values of the protein complex. For new protein *e* in dynamic PPI *cl*_*i*_, the position is updated by following Eq. (10).

10 $${p}_{new,c{l}_{i},e}={p}_{c{l}_{i},e}+\alpha ({p}_{best,c{l}_{i}}-{p}_{c{l}_{i},e})\times rand$$

where ${p}_{new,c{l}_{i},e}$ is the new position of protein *e* in dynamic PPI *cl*_*i*_ and ${p}_{c{l}_{i},e}$ denotes the position in previous generation. ${p}_{best,c{l}_{i}}$ signifies dynamic PPI *cl*_*i*_ which has the best fitness, *α* is the scale factor that determines the influence of best fitness and *rand* is the random variable employed to enhance the diversity of the populations and defined in the range (0, 1).

The movement of a protein *e* for best fitness can be updated using Eq. (11).

11 $${p}_{best,c{l}_{i},e}=\beta \times {p}_{center,c{l}_{i}}$$

where *β* belongs to (0, 1) which is a scale to regulate the effect of ${p}_{center,c{l}_{i}}$ on ${p}_{best,c{l}_{i},e}$. ${p}_{center,c{l}_{i}}$ is the centre of dynamic PPI *cl*_*i*_ and for the *di*^*th*^ dimension it can be computed using the Eq. (12).

12 $${p}_{center,c{l}_{i},d}=\frac{1}{{n}_{c{l}_{i}}}\times \sum _{e=1}^{{n}_{c{l}_{i}}}{p}_{c{l}_{i},e,d}$$

where 1 ≤ *di* ≤ *D*, denotes the *di*^*th*^ dimension and *D* is its total dimension. ${n}_{c{l}_{i}}$ is the number of proteins in dynamic PPI *cl*_*i*_, ${p}_{c{l}_{i},e,d}$ is the *di*^*th*^ dimension of the protein in ${p}_{c{l}_{i},e}$. The centre of the dynamic PPI *cl*_*i*_ is computed through *DI* computations using Eq. (12). The pseudocode for the dynamic PPI updating operator is depicted in Algorithm 1.

**Algorithm 1:**
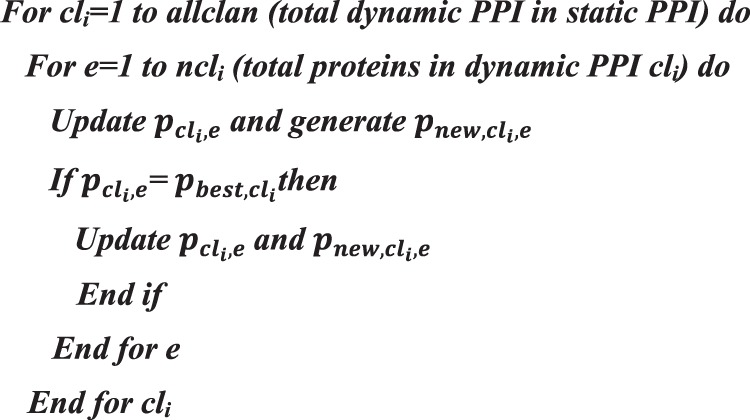
Pseudocode for Clan Updating Operator.

#### Clan separating operator

To enhance the search capacity of the proposed method, the unclustered proteins and clusters with the lowest fitness will exit in every generation as given in Eq (13)^19^.

13 $${p}_{worst,c{l}_{i}}={p}_{min}+({p}_{max}-{p}_{min}+1)\times rand$$

where *p*_*max*_ and *p*_*min*_ are the upper and lower bound of the single protein. ${p}_{worst,c{l}_{i}}$ is the protein or complex with the lowest fitness. The *rand* is the random variable that has stochastic and uniform distribution in the range (0, 1). The pseudocode for the clan separating operator is given in Algorithm 2.

**Algorithm 2:**
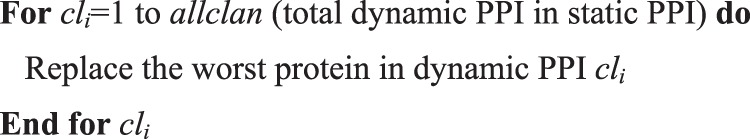
Pseudocode for Clan Separating Operator.

Depending on the clan updating and separating operator, the module of the proposed algorithm is framed as given in Algorithm 3.

**Algorithm 3:**
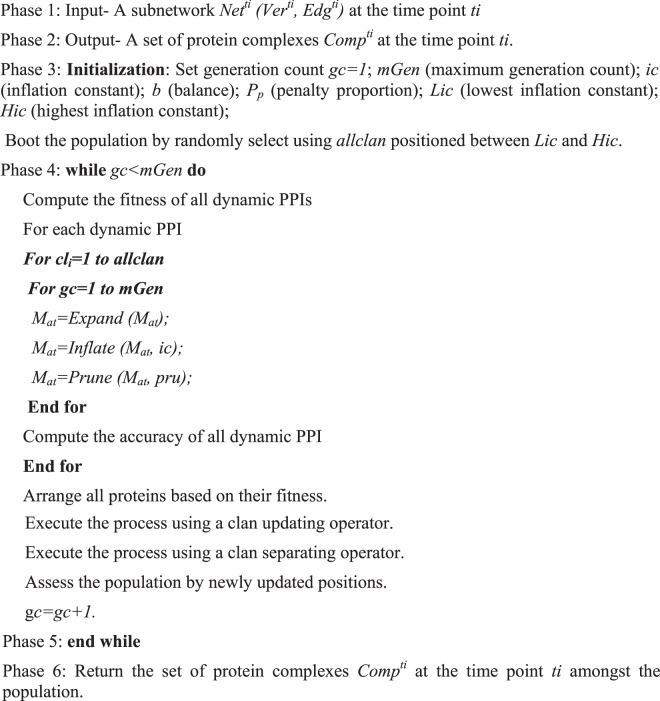
Pseudocode for the Proposed Method.

The relationship between the DMCL-EHO and the protein complex is given in the Table 1.

**Table 1:** The association between the components of DMCL-EHO and the protein complex

DMCL-EHO	Protein Complex
Elephant	The temporary proteins in dynamic subnetwork.
Population	Static PPI Network
Clan	Dynamic PPI Network
Fitness of Clan	Clustering result of the proposed method
Fittest Clan	Best result of the proposed method
Position of an elephant	Value of Parameters

## Experimental Results

### Datasets

In this experiment, the datasets which consists of interactions for both *Saccharomyces cerevisiae* and *Homo Sapiens* are DIP^21^, BioGRID^22^ and STRING^23^. The benchmark PPI datasets employed only for *Saccharomyces cerevisiae* are Gavin2 and Gavin6^24^, Krogan-core and Krogan-extended^25^, Collins^26^, and WI-PHI^27^. The Gavin + Krogan dataset was generated by merging Gavin and Krogan Core datasets. The PPI datasets employed only for *Homo Sapiens* are HPRD^28^, HPID^29^ and PIPs^30^. Table 2 shows the list of datasets used in this experiment.

**Table 2:** List of datasets and gold standard benchmark databases.

S. No	Saccharomyces cerevisiae			Homo Sapiens			Saccharomyces cerevisiae & Homo Sapiens			
	Dataset	No of Proteins	No of Interactions	Dataset	No of Proteins	No of Interactions	Dataset	ORGANISM	No of Proteins	No of Interactions
1	Gavin2	1430	6531	HPRD	10080	39209	DIP	Yeast	5221	24918
								Human	5048	9141
2	Gavin6	1855	7669	HPID	27049	16390	BioGRID	Yeast	7161	53791
								Human	23373	365293
3	Krogan-Core	2708	7123	PIPs	32179	14979	STRING	Yeast	6691	184596
								Human	19566	1258291
4	Krogan-Extended	3581	14076	—	—	—	—	—	—	—
5	Collins	1622	9074	—	—	—	—	—	—	—
6	Gavin + Krogan	2964	13507	—	—	—	—	—	—	—
7	WI-PHI	5955	50000	—	—	—	—	—	—	—
				—	—	—	—	—	—	—
**Gold Standard Databases**										
**S. No**	**Standard Database**			**Number of Proteins**			**Number of Interactions**		**Number of Complexes**	
1	CYC2008			1627			408		408	
2	MIPS			1189			11119		203	
3	SGD			1279			19854		323	
4	PCDq			9268			32198		1264	

The gene expression data used in this study for *Saccharomyces cerevisiae* (*GSE3431*)^31^ and *Homo Sapiens* (GSE3933)^32^ are taken from the GEO database.

The predicted complexes are compared to gold standard benchmark databases such as CYC2008^33^, MIPS^34^, SGD^35^ for *Saccharomyces cerevisiae* organism and PCDq^36^ benchmark dataset for *Homo sapiens* organism. The percentage of overlapping interactions among the datasets in Gavin2 is 32%, Gavin6 is 53%, Krogan-core is 46%, Collins is 56%, HPRD is 23%, PIPs is 57%, DIP is 2%, BioGRID is 55% and STRING is 47%^37,38^.

### Performance measures

To evaluate and compare the clustering results of predicted protein complexes, the generated complexes were compared and matched with the gold standard benchmark protein complexes. Assume P_r_(V_Pr_,E_Pr_) and B_e_(V_Be_,E_Be_) be the set of vertices (proteins) and edges (interaction) of a predicted protein complex and benchmark protein complexes.

#### Complex similarity score (CSS)

CSS is defined as the closeness of two protein complexes namely predicted (P_r_) and benchmark (B_e_) protein complexes and they are computed based on Eq. 14.

14 $$CSS({P}_{r},{B}_{e})=\frac{|{V}_{Pr}\,\,{V}_{Be}{|}^{2}}{|{V}_{Pr}|\ast |{V}_{Be}|}$$

where *V*_*pr*_ and *V*_*Be*_ denotes the set of proteins in predicted and benchmark protein complexes. If *CSS*(*P*_*r*_, *B*_*e*_) is equal to 0, it denotes that the predicted and benchmark protein complexes do not have any common protein complexes. On the contradictory, if *CSS*(*P*_*r*_, *B*_*e*_) is equal to 1, then the predicted complex P_r_(V_Pr_, E_Pr_) has the same equal nodes as the benchmark complex B_e_(V_Be_, E_Be_). Here, if *CSS*(*P*_*r*_, *B*_*e*_) > 0.2, it is considered as the predicted and benchmark protein complexes are identical^39^.

Now, to assess the performance of predicted protein clusters, four commonly employed measures are utilized such as Precision, Recall, F-Measure, Coverage Ratio and Accuracy.

#### Precision

Precision is defined as the accuracy of predicted protein complexes that are identical to the benchmark protein complexes. If the precision value is high, it indicates that the predicted complexes are likely to be true positive. The precision of the protein complexes is computed based on Eq. (15).

15 $$Precision=\frac{{N}_{Pc}}{|Predicte{d}_{set}|}$$

#### Recall

Recall is defined as the accuracy of benchmark protein complexes that are identical to the predicted complexes. If the recall value is high, it indicates that the predicted complex has a good number of coverage of the proteins in the gold standard complexes. The recall of the protein complexes is computed based on Eq. (16).

16 $$Recall=\frac{{N}_{Bc}}{|Know{n}_{set}|}$$

where *N*_*Pc*_ is denoted as the number of predicted complexes which match at least one recognized benchmark complex, *N*_*Bc*_ is denoted as the number of recognised benchmark complexes which match at least one predicted complex, *Predicted*_*set*_ is denoted as the set of complexes predicted by the proposed approach and *Known*_*set*_ is denoted as the set of recognised gold standard benchmark protein complexes.

#### Coverage ratio (CR)

CR is defined as the fraction of proteins in benchmark complex *V*_*Be*_ found in predicted complex *V*_*pr*_ and they are computed based on Eq. (17).

17 $$CR=\frac{\sum _{i}{\max }\,{T}_{i,j}}{{\sum }_{i}|{V}_{Be}|}$$

where *V*_*Be*_ is denoted as the set of proteins in benchmark protein complexes. *T*_*i*, *j*_ is denoted as the common number of proteins between *V*_*pr*_ and *V*_*Be*_.

#### F-Measure

F-Measure is defined as the harmonic mean, i.e., a rational mixture of both precision and recall and it is computed based on Eq. (18).

18 $$F-Measure=\frac{2\,(Precision\ast Recall)}{(Precision+Recall)}$$

#### Accuracy

Accuracy is defined as the geometrical mean i.e the trade-off between precision and recall and it is computed based on Eq. (19).

19 $$Accuracy=\sqrt{Precision\ast Recall}$$

#### Number of Clusters

The number of clusters is defined as the total quantity of clusters formed from the PPI network after the clustering process has been completed.

**Figure 2:**
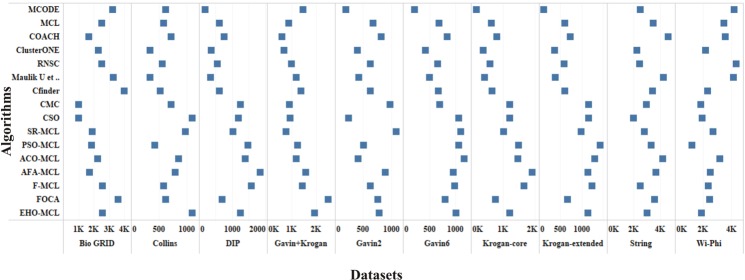
Comparison of Number of Clusters with various Datasets and Algorithms against CYC2008 Benchmark Dataset,

**Figure 3:**
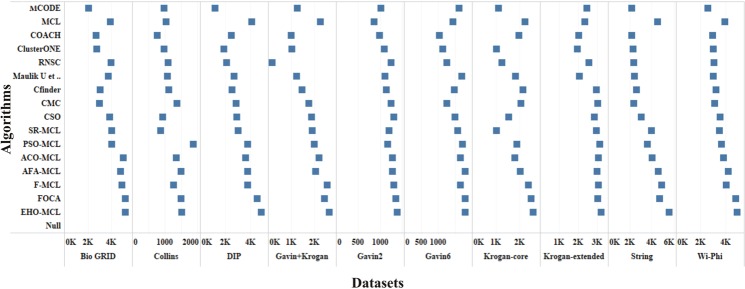
Comparison of Coverage Ratio with various Datasets and Algorithms against CYC2008 Benchmark Dataset.

**Figure 4:**
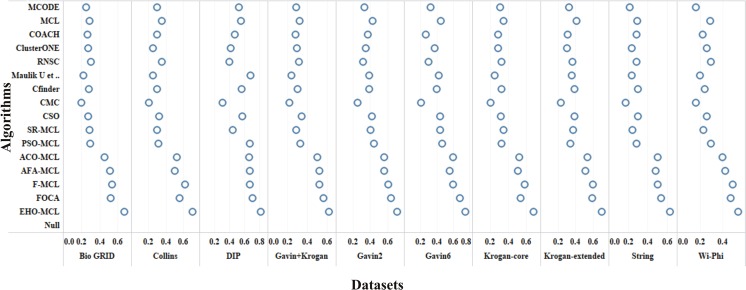
Comparison of Precision with various Datasets and Algorithms against CYC2008 Benchmark Dataset.

**Figure 5:**
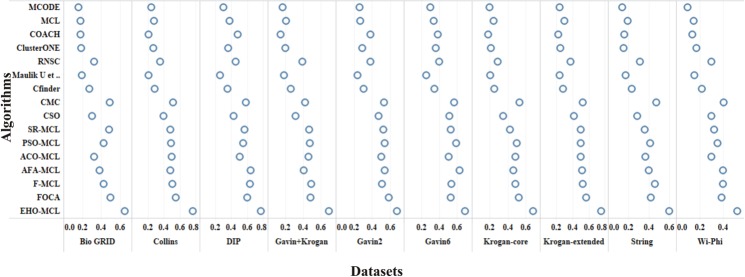
Comparison of Recall with various Datasets and Algorithms against CYC2008 Benchmark Dataset.

**Figure 6:**
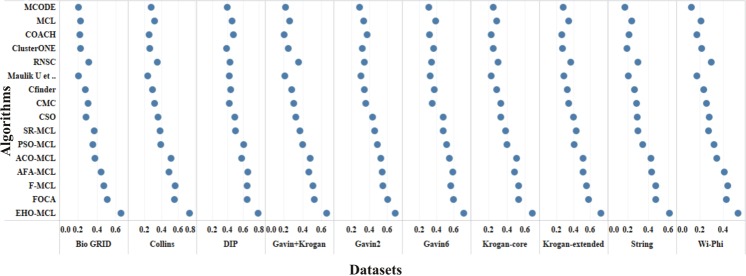
Comparison of F-Measure with various Datasets and Algorithms against CYC2008 Benchmark Dataset.

**Figure 7:**
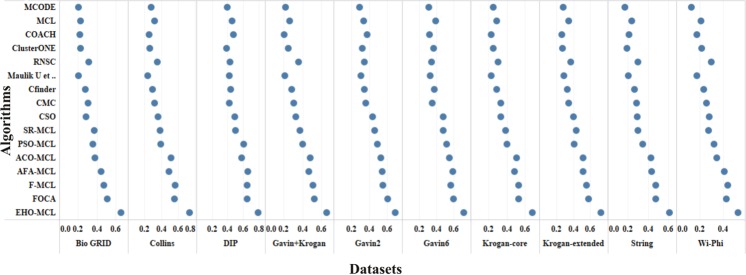
Comparison of Accuracy with various Datasets and Algorithms against CYC2008 Benchmark Dataset.

**Figure 8:**
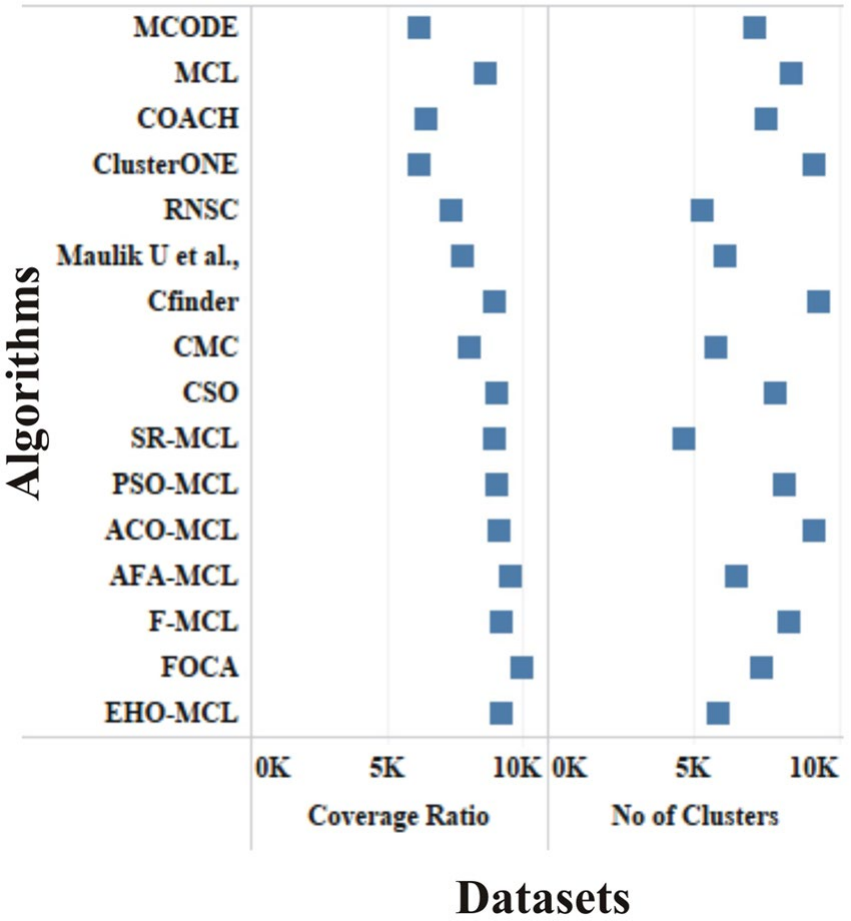
Comparison of Number of Clusters and Coverage Ratio with HPRD Dataset and Algorithms against PCDq Benchmark Dataset.

**Figure 9:**
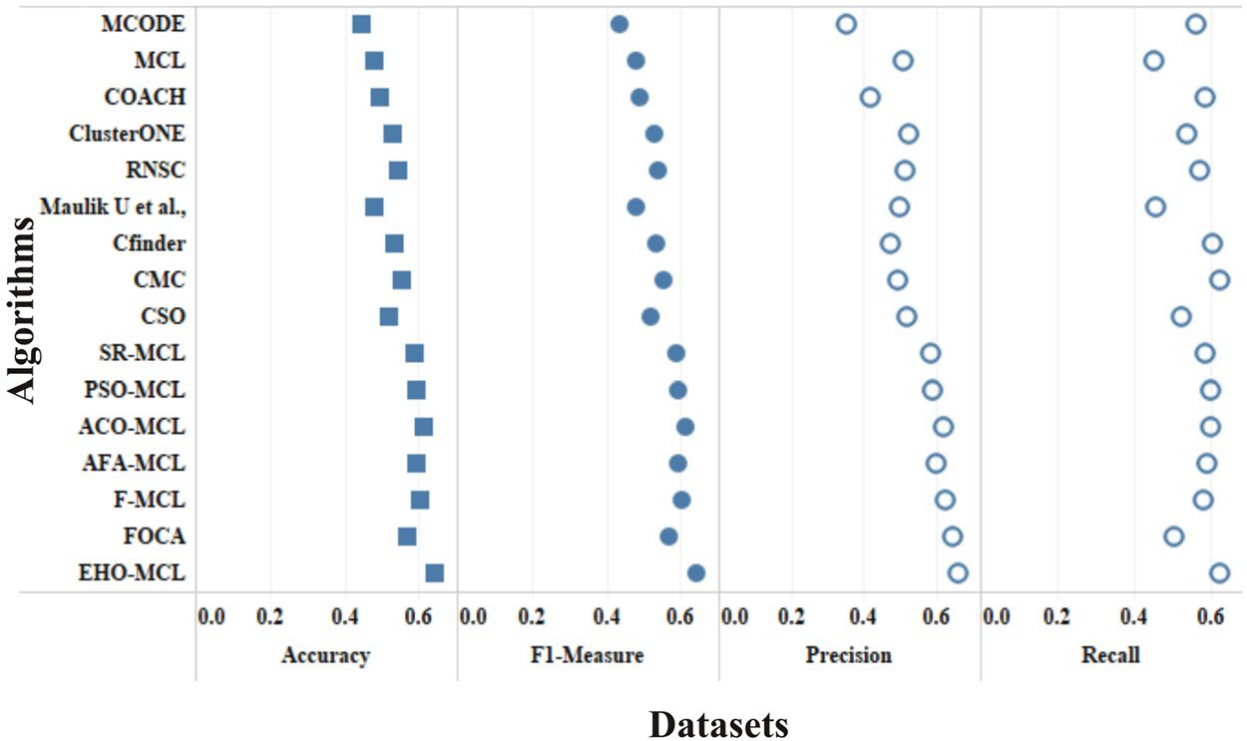
Comparison of Precision, Recall, F-Measure and Accuracy with HPRD Dataset and Algorithms against PCDq Benchmark Dataset.

The performance measures such as coverage ratio, the number of clusters, precision, recall, f-measure and accuracy of the proposed method for *Saccharomyces cerevisiae* are compared with various datasets and existing algorithms against CYC2008 benchmark database and the graphical representation of the comparison is depicted in Figs 2–7. Also, the performance measures such as coverage ratio, the number of clusters, precision, recall, f-measure and accuracy of the proposed method for *Homo sapiens* are compared with various datasets and existing algorithms against PCDq benchmark database and the graphical representation of the comparison is depicted in Figs 8 and 9. The comparison of performance measures for the proposed method with various datasets and existing algorithms against the MIPS and SGD benchmark database for *Saccharomyces cerevisiae* is given in supplementary material.

From Figs 2 and 8, it is inferred that the number of clusters in the proposed method is less when compared to FOCA, AFA-MCL and ACO-MCL as they try to get solution from all the proteins in the network. These methods will not discard the undesirable proteins which may result in false positives. But in the proposed method, the clusters which has less than three proteins are discarded. Hence the precision, recall, F-Measure and accuracy are high for the proposed method.

From Figs 3 and 8, it is observed that the proposed method has more coverage ratio than the existing methods since it employs the iterated clustering approach. This enhances the coverage of proteins in the network as the proteins in the benchmark complexes are highly found in the predicted complexes. From Figs 4–7 and 9 it is observed that the precision, recall, F-Measure and accuracy shows fluctuations for PSO-MCL, ACO-MCL, AFA-MCL, F-MCl, FOCA and EHO-MCL. The mean of these measures for all the datasets shows that the proposed method performs better than the existing methods because it has employed the dynamic PPI along with EHO.

## Implementation and Discussion

The computational issue of attaining a solution with a high accuracy solution for protein complex detection from dynamic PPI is still a challenging task. In this paper, the elephant herd optimization algorithm along with Markov clustering technique is combined to solve the protein complex detection problem. The proposed method provides an enhancement of the results compared to all the other popular existing methods. This work was executed on 2.00 GHz Intel i3 with 8GB of memory running on Windows 10.

The number of clusters is small in an average when compared to other existing methods, due to the deletion of proteins without interactions. Here, the minimum number of proteins inside a cluster should be three or more and that are considered as a protein complex. The protein cluster with less than three proteins are removed. The proposed method was evaluated based on the removal of noise, insertion and deletion of random protein interactions, large PPI network, namely WI-PHI, various parameter analysis, statistical significance and finally with biological significance.

### Evaluation by noise removal

The PPI networks are obtained from high-throughput experiments, the large coverage of the PPI network comprises of noise in the format of false positive interactions and redundant data. The main challenge of clustering these PPI networks is present in the PPI networks itself. In this method, after the clustering process is accomplished, the proteins that do not present in any of the clusters is also considered as a noise. These solitary proteins that do not interact with any other proteins will not provide any valuable information. The minimum number of proteins inside the cluster is set to be three in this work. Thus, the isolated proteins and clusters with below three proteins are considered as a noise and they are removed by the clan separating operator by the elephant herd optimization method. Many evolutionary approaches are inheriting the undesirable proteins from one generation to another which may lead to loss of accuracy, but EHO approach will discard the undesirable proteins from the population in the clan separating operator that leads to the optimal solution. The comparison of EHO with other existing methods is depicted in Figs 2–9.

### Evaluation by adding and removing random protein interactions

**Figure 10:**
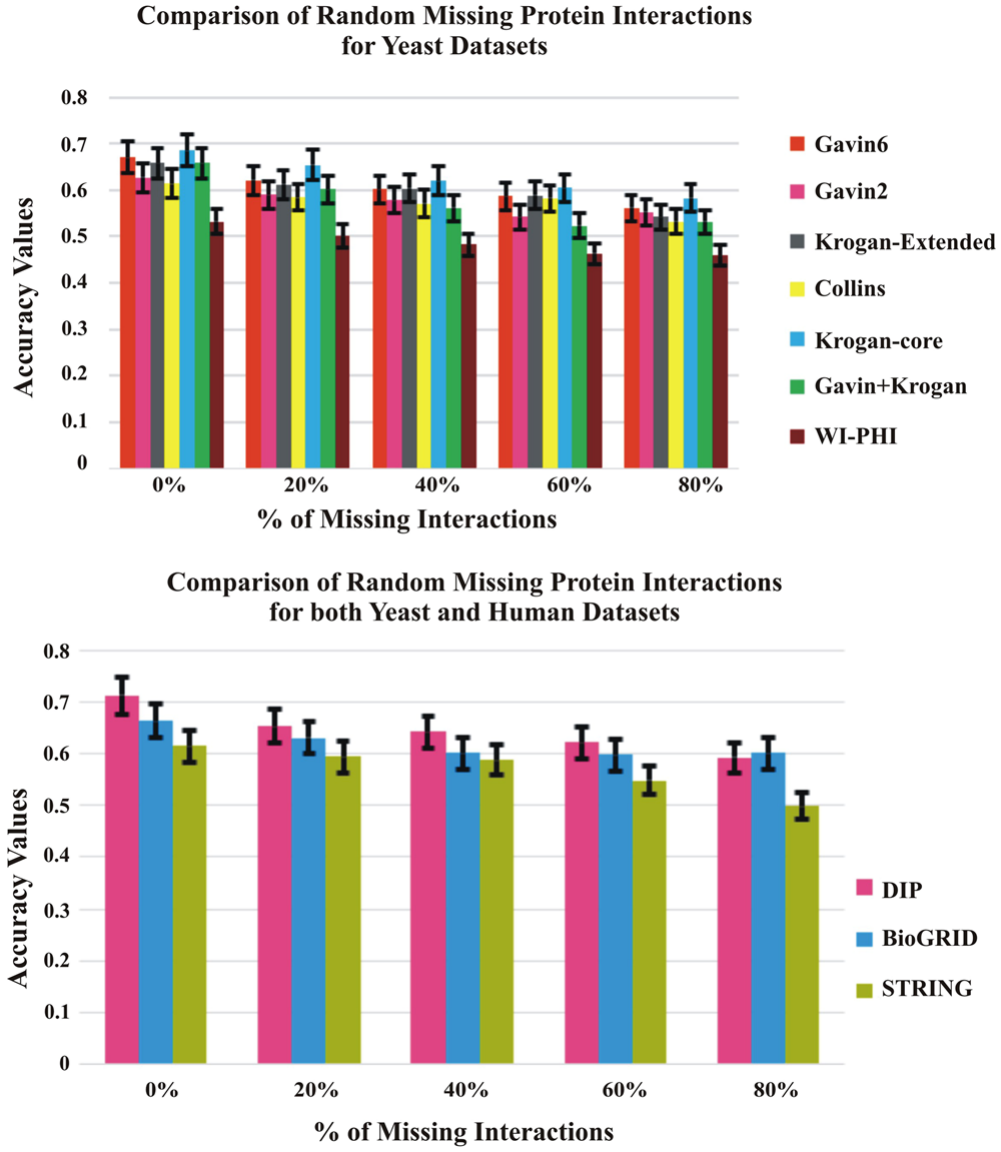
Comparison of Accuracy with Random Deletion of Protein Interactions on DIP Dataset against CYC2008 benchmark database.

**Figure 11:**
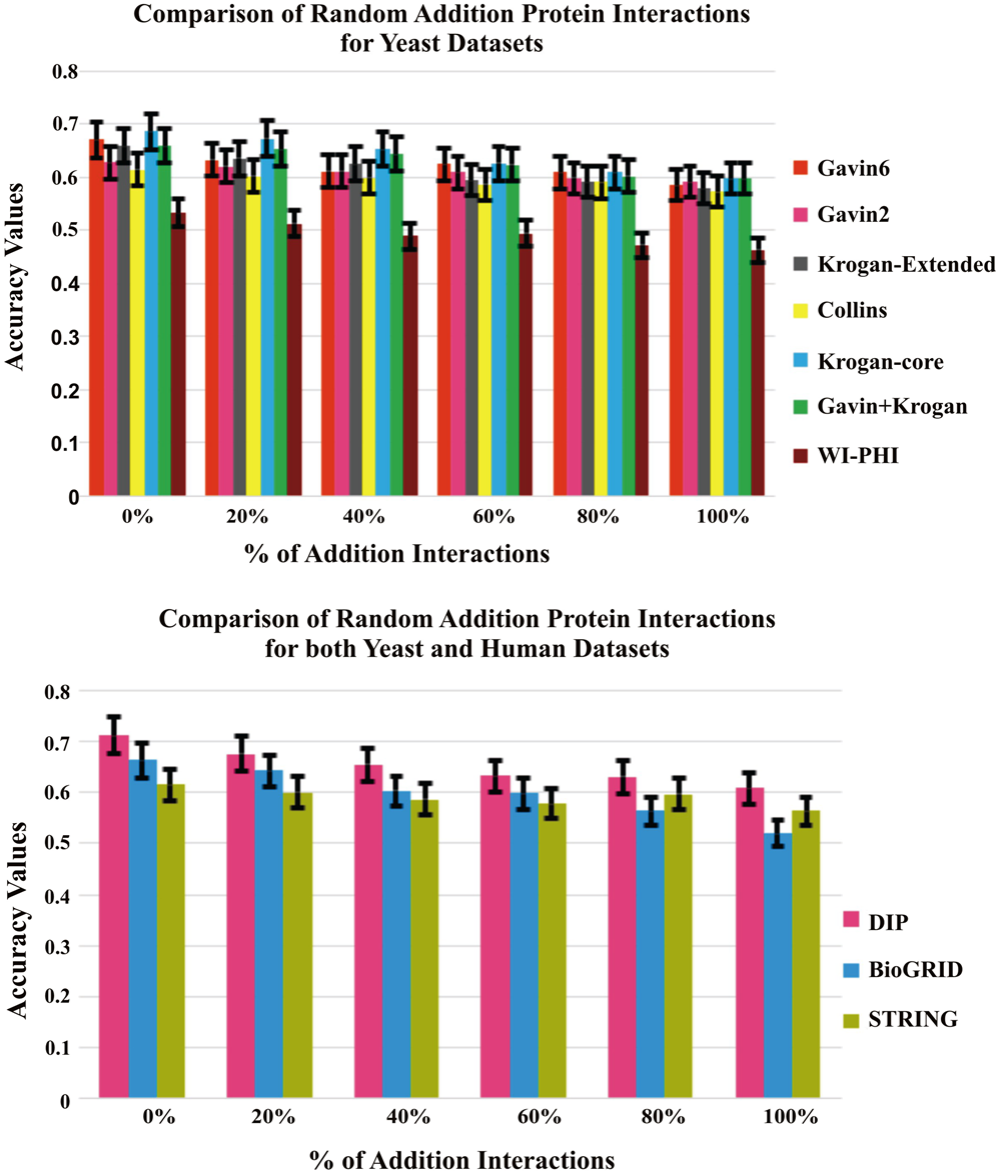
Comparison of Accuracy with Random Insertion of Protein Interactions on DIP Dataset against CYC2008 benchmark database.

The testing of the proposed method is accomplished by inserting and deleting the random interactions of the PPI network to evaluate its performance. The noise can also be any missing information (false negatives) or added noise (false positives) in the PPI network. The DIP dataset is used for evaluation of adding and removing random interactions. The missing information of PPI network is processed by removing the proportion of edges randomly (0%, 20%, 40%, 60%, 80%) and the false positive information of PPI network is processed by adding the proportion of edges randomly (0%, 20%, 40%, 60%, 80%, 100%). The performance of the proposed method by adding and removing the random interactions are depicted in Figs 10 and 11.

From the Figs 10 and 11, it is observed that even though the random insertions and deletion of the protein interactions are employed on the dataset, the proposed method performs better than other existing approaches.

### Evaluation by large PPI network WI-PHI dataset

In addition to analyse the performance of the proposed method on the large PPI dataset, WI-PHI^27^ dataset of *Saccharomyces cerevisiae* was employed which comprises of 5955 proteins and 50,000 protein interactions. The proposed method and also the existing methods were executed on this large dataset and compared the predicted clusters with the various gold standard benchmark databases. The comparison of the existing and proposed method on WI-PHI dataset is depicted in Figs 2–7.

### Evaluation by parameter analysis

Generally, every metaheuristic approach is based on certain stochastic dissemination. Hence, diverse runs will produce various diverse results. This work implements 500 independent runs in order to score optimal solution. In general, 20 numbers of clans were employed as per literature. The execution process will be terminated, if the best result generated in each iteration remains interchangeable for 100 successive iterations or the maximum number of generations is attained. The assignment of parameter values was adjusted based on the experimental results. It was identified that the parameters of the proposed method that has values of α = 0.5 and β = 0.1 produced better solution among different values and hence were allocated. It was observed that the optimal solution was identified after 315^th^ generation. For all the performance measures, there were fluctuations during the first 10 runs of the experiment and in the future runs reliability was observed. Figures 2–9 shows the average outcome of performance measures for the above parameter values of the proposed method. Table 3 shows the various parameter values for the proposed approach and the other existing approaches of protein complex detection.

**Table 3:** Various Parameter Values of proposed and existing methods for protein complex detection.

Parameters	Variable	MCL	SR-MCL	CSO	PSO-MCL	ACO-MCL	AFA-MCL	F-MCL	FOCA	EHO-MCL
Inflation constant	ic	2	2	automatic	automatic	automatic	automatic	automatic	automatic	Automatic
Lowest ic	Lic			1	1	1	1	1	1	1
Highest ic	Hic			6	6	6	6	6	6	6
Balance	B	0.5	0.5	0.5	0.5	0.5	0.5	0.5	0.5	0.5
Penalty proportion	P_p_	1.25	1.25	1.25	1.25	1.25	1.25	1.25	1.25	1.25
Number of population	K		10	10	10	10	10	10	10	10
Maximum geneartion	mGen		5	5	5	5	5	5	5	5
Cognitive and Social acceleration coefficient	C1 and c2				2					
Maximum velocity	Max_V_				0.5					
evaporation rate	р					0.1				
Heuristic information	H					1.2				
pseudo random proportion selection rule	q0					0.9				
Visual range	Vis						0.9			
Step length	S						0.05	0.05	0.05	
Light absorption coefficient	λ							1.0		
Maximum attractiveness	Ma							1.0		
Scale regulates ${p}_{c{l}_{i},e}$	α									0.5
Scale regulates ${p}_{center,c{l}_{i}}$	β									0.1
Number of clans	allclan									20

### Evaluation by statistical significance

The proposed method was also assessed by utilizing non-parametric test such as, Wilcoxon Matched-Pair Signed-Rank Test among each pair of approaches that produces the statistical consequence. The discrepancy between the F-Measure and Accuracy for every entry in Figs 6 and 7 was tested based on the confidence level of 1% (p-value < 0.01). The p-value less than 0.01 are assumed as highly significant and the values greater than 0.01 are assumed as insignificant values. The scores of F-Measure and Accuracy is alone considered as they are computed based on precision and recall. The Statistical Significance of the proposed and existing approaches based on F-Measure and Accuracy is depicted in Table 4. The scores of upper right positions of the table are attained from F-Measure of proposed and various existing algorithms based on DIP dataset against CYC2008 benchmark database. The scores of lower left positions of the table are attained from Accuracy of proposed and various existing algorithms based on DIP dataset against CYC2008 benchmark database. From Table 4, it is shown that the proposed method is statistically significant in nature compared to all the existing methods.

**Table 4:** Statistical Significance of proposed and existing approaches based on F-Measure and Accuracy.

	MCODE	MCL	COACH	ClusterONE	RNSC	Maulik U *et al*.^37^	CFinder	CMC	CSO	SR-MCL	PSO-MCL	ACO-MCL	AFA-MCL	F-MCL	FOCA	EHO-MCL
MCODE	0	0.005	0.241	0.028	0.015	0.508	0.012	0.017	0.019	0.026	0.018	0.006	0.006	0.006	0.005	0.004
MCL	0.005	0	0.047	0.022	0.059	0.035	0.333	0.878	0.52	0.008	0.007	0.008	0.006	0.006	0.006	0.005
COACH	0.241	0.037	0	0.646	0.022	0.508	0.013	0.093	0.008	0.009	0.009	0.005	0.006	0.007	0.005	0.005
ClusterONE	0.174	0.089	0.521	0	0.089	0.017	0.015	0.059	0.025	0.012	0.008	0.008	0.007	0.008	0.005	0.005
RNSC	0.05	0.074	0.022	0.022	0	0.025	0.022	0.015	0.016	0.028	0.008	0.007	0.007	0.009	0.005	0.005
Maulik U *et al*.^37^	0.333	0.065	0.103	0.035	0.005	0	0.035	0.027	0.025	0.015	0.005	0.006	0.006	0.006	0.005	0.003
CFinder	0.024	0.138	0.013	0.029	0.028	0.035	0	0.285	0.025	0.015	0.005	0.005	0.005	0.008	0.005	0.002
CMC	0.035	0.093	0.017	0.022	0.203	0.03	0.059	0	0.015	0.025	0.015	0.005	0.005	0.009	0.005	0.001
CSO	0.038	0.045	0.015	0.019	0.169	0.027	0.025	0.017	0	0.028	0.012	0.004	0.005	0.006	0.005	0.003
SR-MCL	0.027	0.035	0.035	0.025	0.017	0.021	0.035	0.015	0.013	0	0.022	0.005	0.004	0.005	0.005	0.004
PSO-MCL	0.023	0.028	0.018	0.015	0.005	0.019	0.012	0.011	0.009	0.018	0	0.013	0.004	0.004	0.004	0.004
ACO-MCL	0.015	0.019	0.013	0.01	0.005	0.015	0.011	0.005	0.009	0.008	0.013	0	0.016	0.003	0.005	0.003
AFA-MCL	0.012	0.002	0.01	0.009	0.005	0.01	0.005	0.009	0.005	0.009	0.008	0.139	0	0.017	0.009	0.002
F-MCL	0.009	0.005	0.009	0.005	0.005	0.005	0.005	0.007	0.006	0.004	0.005	0.005	0.007	0	0.003	0.003
FOCA	0.004	0.007	0.006	0.002	0.005	0.004	0.005	0.005	0.005	0.005	0.004	0.005	0.005	0.005	0	0.002
EHO-MCL	0.005	0.004	0.004	0.003	0.002	0.005	0.002	0.008	0.002	0.003	0.003	0.002	0.004	0.005	0.001	0

### Evaluation by biological significance

Many of the existing methods solve the protein complex detection problem based on the topological similarity. But to obtain some useful biological information, the computational methods should be biologically significant in nature. This proposed method is evaluated in the biological significance test. The gold standard benchmark databases are manually annotated based on the information from biologically experimental analysis. Thus, the detected protein complexes obtained from the proposed method is compared and matched with the benchmark databases. Few benchmark databases such as CYC2008, MIPS, SGD databases for *Saccharomyces cerevisiae* and the PCDq database for Homo sapiens are employed for assessing the proposed method. Table 5 displays the common protein complexes between the CYC2008 benchmark database and the proposed method for DIP and Krogan-extended. Also, the common biological process, molecular function and the cellular component of the obtained protein complexes are displayed. Correspondingly, the common pathway annotations of the predicted protein complexes are obtained from the KEGG database are displayed.

**Table 5:** Top 5 Common Protein Complexes, Gene Ontology Functions and KEGG Pathways of the Predicted Complexes of proposed method.

S.no	Complex name	Real Complexes	Correctly Predicted Complexes	Wrong Complexes	BP	MF	CC	Pathways
**Krogan-extended**								
1	Paf1p complex	YOL145C, YLR418C, YBR279W, YML069W, YGL207W, YGL244W,	YOL145C, YLR418C, YBR279W, YGL244W,	YEL037C	Positive regulation of transcription elongation from RNA polymerase I promoter (GO:2001209) **P-Value:** 1.4E-9 **Enrichment Score:** 5.3E-8	RNA polymerase II C-terminal domain phosphoserine binding (GO:1990269) **P-Value:** 3.5E-9 **Enrichment Score:** 6.6E-8	Cdc73/Paf1 complex(GO:0016593) **P-Value:** 2.9E-8 **Enrichment Score:** 2.8E-7	NIL
2	Condensin complex	YFR031C, YLR086W, YDR325W, YBL097W, YNL088W,	YFR031C, YLR086W, YDR325W, YBL097W, YNL088W,	NIL	tRNA gene clustering (GO:0070058) **P-Value:** 1.3E-3 **Enrichment Score:** 1.7E-2	Chromatin binding (GO:0003682) **P-Value:** 2.1E-2 **Enrichment Score:** 1.4E-1	Condensed nuclear chromosome(GO:0000794) **P-Value:** 2.2E-2 **Enrichment Score:** 4.4E-2	Cell- Cycle Yeast **P-Value:** 5.6E-2 **Enrichment Score:** 5.6E-2
3	RNA polymerase II mediator complexX	YHR058C, YDR308C, YHR041C, YNR010W, YOL135C, YBR253W, YOR174W, YMR112C, YPR168W	YHR058C, YDR308C, YHR041C, YBR253W, YOR174W, YMR112C, YPR168W	YKL081W, YCR033W	Positive regulation of transcription from RNA polymerase II promoter (GO:0045944) **P-Value:** 3.4E-7 **Enrichment Score:** 1.5E-6	transcription factor activity, RNA polymerase II transcription factor binding (GO:0001076) **P-Value:** 5.3E-12 **Enrichment Score:** 6.3E-11	Mediator complex (GO:0016592) **P-Value:** 4.1E-15 **Enrichment Score:** 6.2E-15	NIL
4	RNA polymerase I subunit	YNL248C, YJR063W, YJL148W, YOR340C, YPR010C, YPR187W, YBR154C, YOR224C, YNL113W	YNL248C, YJR063W, YJL148W, YOR340C, YPR010C, YPR187W, YBR154C, YNL113W	YIL7095W	Ribosome biogenesis (GO:0042254) **P-Value**: 7.1E-10 **Enrichment Score:** 2.8E1	DNA-directed RNA polymerase activity (GO:0003899) **P-Value:** 2.3E-14 **Enrichment Score:** 3.6E2	DNA-directed RNA polymerase I complex (GO:0005736) **P-Value:** 5.3E-17 **Enrichment Score:** 1.5E2	RNA polymerase, **P-Value:** 4.0E-12 **Enrichment Score:** 6.2E1 Pyrimidine metabolism, **P-Value:** 8.8E-10 **Enrichment Score:** 2.7E1 Purine metabolism, **P-Value:** 6.2E-9 **Enrichment Score:** 2.0E1 Metabolic pathways **P-Value:** 9.1E-4 **Enrichment Score:** 2.7E0
5	Small Subunit (SSU) processome complexes	YLR409C, YLR222C, YJL069C, YDR398W, YGR128C, YJL109C, YDR324C, YDR449C, YDL148C, YLR129W	YLR409C, YLR222C, YJL069C, YDR398W, YGR128C, YJL109C, YDR324C, YDR449C, YDL148C, YLR129W	NIL	Ribosomal small subunit biogenesis (GO:0042274) **P-Value:** 3.7E-2 **Enrichment Score:** 5.2E-2	snoRNA binding(GO:0030515) **P-Value:** 1.3E-9 **Enrichment Score:** 3.8E-9	Small-subunit processome (GO:0032040) **P-Value:** 2.8E-15 **Enrichment Score:** 4.0E-14	Ribosome biogenesis in eukaryotes **P-Value:** 3.9E-9 **Enrichment Score:** 3.9E-9
**DIP**								
1	NOT core complex	YDL165W, YCR093W, YAL021C, YIL038C, YGR134W, YNL288W, YDR252W, YER068W, YNR052C, YPR072W	YDL165W, YCR093W, YAL021C, YIL038C, YDR252W, YER068W, YNR052C, YPR072W	YMR149WYJR035W, YJR112W	Nuclear-transcribed mRNA catabolic process, deadenylation-dependent decay (GO:0000288) **P-Value:** 6.0E-14 **Enrichment Score:** 1.6E-12	NIL	CCR4-NOT core complex (GO:0030015) **P-Value:** 2.6E-21 **Enrichment Score:** 1.8E-20	RNA degradation **P-Value:** 3.7E-10 **Enrichment Score:** 3.7E-10
2	Mitochondrial F1F0 ATP synthase	YLR295C, YDL004W, YBL099W, YBR039W, YJR121W, YPL078C, YKL016C, YEL027W, YEL051W, YDL185W, YLR447C	YLR295C, YDL004W, YBL099W, YBR039W, YJR121W, YPL078C, YKL016C, YEL027W, YEL051W,	YNL189W, YER031C, YGL181W	ATP hydrolysis coupled proton transport (GO:0015991) **P-Value:** 2.2E-4 **Enrichment Score:** 6.8E-4	Proton-transporting ATPase activity, rotational mechanism (GO:0046961) **P-Value:** 8.0E-14 **Enrichment Score:** 3.6E-*13	Mitochondrial proton-transporting ATP synthase complex(GO:0005753) **P-Value:** 4.1E-3 **Enrichment Score**: 9.1E-3	Oxidative phosphorylation, P-Value: 9.4E-13 Enrichment Score: 2.8E-12 Metabolic pathways **P-Value:** 8.8E-5 **Enrichment Score:** 1.3E-4
3	Putative ferric reductase	YBR207W, YLR214W, YER145C, YLR047C, YOL152W, YKL220C, YFL041W, YMR319C, YLL051C,	YBR207W, YLR214W, YER145C, YLR047C, YLL051C,	YOR227W, YKL196C,	Iron ion homeostasis (GO:0055072) **P-Value:** 6.0E-10 **Enrichment Score:** 9.6E-9	Ferroxidase activity (GO:0004322) **P-Value:** 1.8E-5 **Enrichment Score:** 1.3E-4	Plasma membrane (GO:0005886) **P-Value:** 2.1E-3 **Enrichment Score:** 2.1E-2	NIL
4	Component of spindle pole body	YKL042W, YDR356W, YPL124W, YMR117C, YAL047C, YHR172W, YNL126W, YML124C, YLR212C, YNL188W	YDR356W, YPL124W, YMR117C, YAL047C, YHR172W, YNL126W, YML124C, YLR212C, YNL188W	YML048W	Microtubule nucleation(GO:0007020) **P-Value:** 1.3E-14 **Enrichment Score:** 3.2E-13	Structural constituent of cytoskeleton(GO:0005200) **P-Value**: 9.5E-16 **Enrichment Score**: 8.0E-15	Microtubule organizing center part(GO:0044450) **P-Value:** 2.7E-3 **Enrichment Score:** 1.1E-2	NIL
5	PRoteinase yscE	YKL206C, YER012W, YJL001W, YFR050C, YMR314W, YOL038W, YBL041W, YML092C, YGR135W, YOR362C, YER094C	YKL206C, YER012W, YJL001W, YFR050C, YOL038W, YBL041W, YML092C, YGR135W, YOR362C, YER094C	YOL061W, YIL006W	Proteasomal ubiquitin-independent protein catabolic process(GO:0010499) **P-Value:** 1.6E-18 **Enrichment Score:** 1.1E-17	Threonine-type endopeptidase activity (GO:0004298) **P-Value:** 2.6E-19 **Enrichment Score:** 1.6E-18	Proteasome storage granule (GO:0034515) **P-Value:** 1.1E-16 **Enrichment Score:** 4.4E-16	Proteasome **P-Value:** 1.4E-13 **Enrichment Score:** 1.4E-13

**Figure 12:**
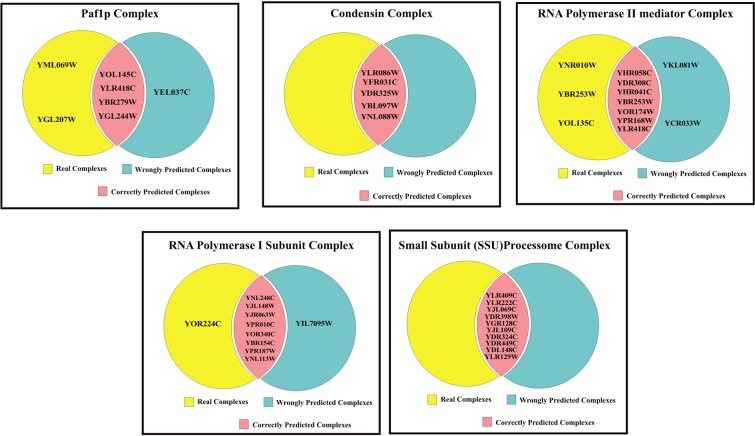
Top 5 common protein complexes, gene ontology functions and KEGG pathways of the predicted complexes of proposed method on Krogan-Extended Dataset.

**Figure 13:**
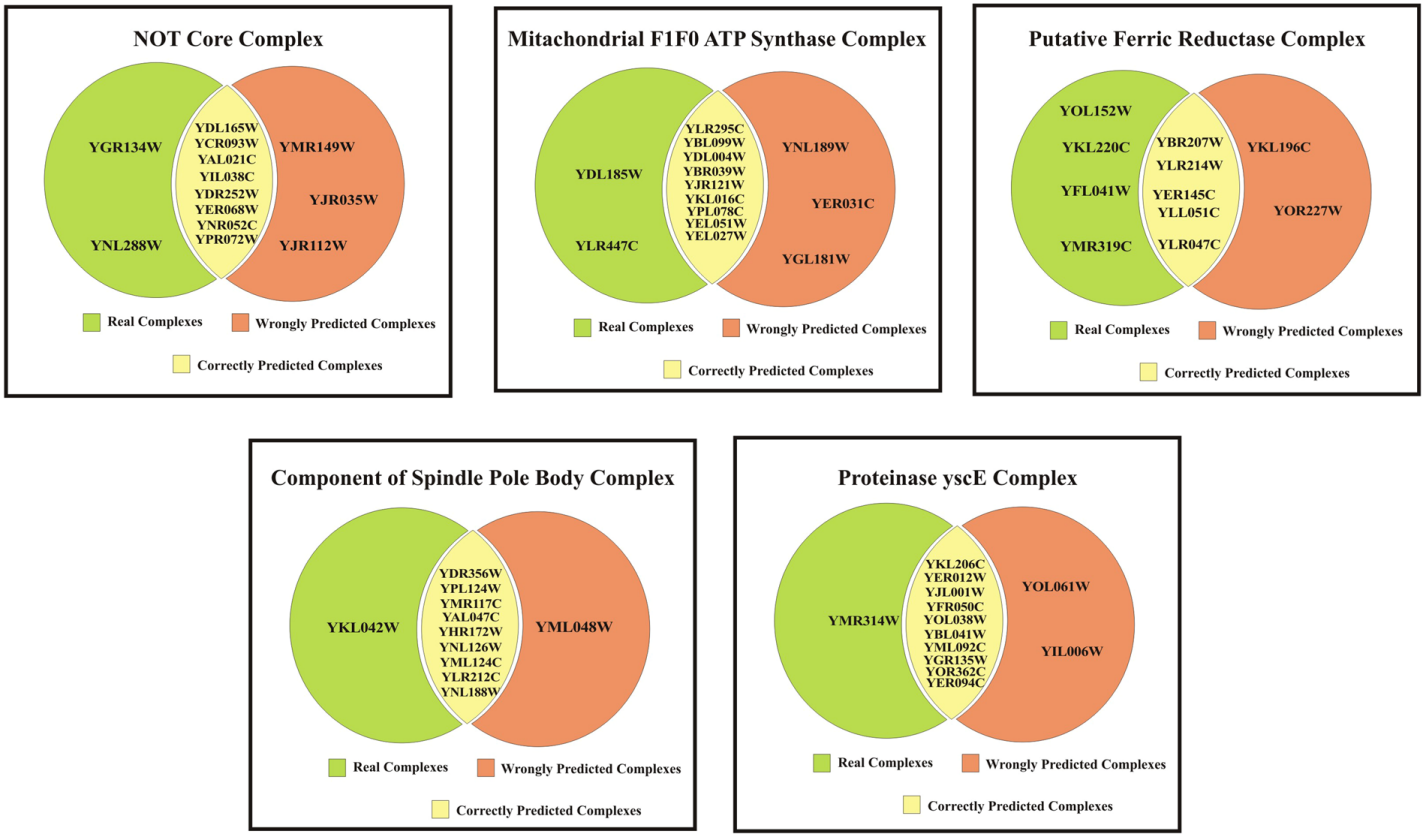
Top 5 common protein complexes, gene ontology functions and KEGG pathways of the predicted complexes of proposed method on DIP Dataset.

The predicted complex gene ontology and KEGG pathway enrichment analysis were predicted by using the DAVID gene function classification online tool. The overall predicted complex enrichment score and the respective gene ontology elements and KEGG pathway enrichment scores are displayed in Table 5. The pictorial representation of the common RNA Polymerase KEGG Pathway of the predicted protein Complex on Krogan-extended dataset and common Oxidative Phosphorylation KEGG Pathway of the predicted protein complex on DIP dataset is exhibited in the supplementary information. The RNA polymerase is essential for nucleolar assembly and for high polymerase loading rate. Oxidative phosphorylation is the metabolic pathway in which cells use enzymes to oxidize nutrients, thereby releasing energy which is used to produce adenosine triphosphate (ATP)^40–42^. The pictorial representation of the Top 5 common protein complexes, gene ontology functions and KEGG pathways of the predicted complexes of proposed method is given as Venn diagram in Figs 12 and 13.

### Execution time

**Figure 14:**
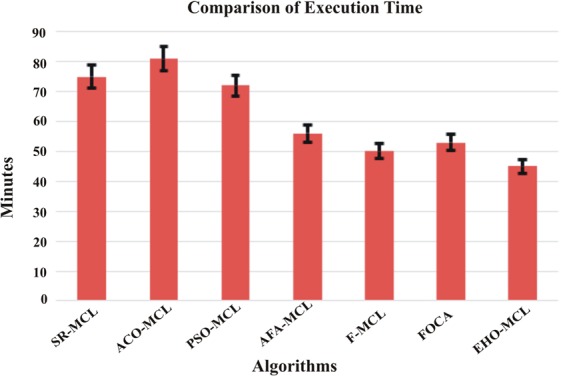
Comparison of Average Execution Time of the proposed algorithm with the existing algorithms.

Besides the accuracy, the time required to detect the dynamic protein complexes is also an important factor. Processing the various benchmark datasets with various numbers of proteins and different interactions requires more time complexity due to stochastic optimization methods. Subsequently, not all methods were available under the same platform, the execution of many of the approaches were done on virtual machines, which prohibited us from accomplishing an exact comparison of their relative execution times. Thus, here the average execution time of SR-MCL, ACO-MCL, PSO-MCL, AFA-MCL, F-MCL, FOCA AND EHO-MCL is displayed in Fig. 14.

From Fig. 14, it is observed that in this research, the proposed algorithm has less execution time when compared to other algorithms, due to the clan separating operator of EHO approach. It is inferred that the proposed EHO-MCL is efficient for detecting dynamic protein complexes. In future, the EHO-MCL can be further optimized in multicore CPU.

## Conclusion

The Protein Complex detection is an exposed problem for scientists. The solution for the complex problem should be recurrently improved as they are important in the analysis of the biological process. The volume of PPI networks has also been increased due to high-throughput experiments, the lack of accurate computational model for protein complex detection exists. Many of the existing researches were employed on the static PPI data that do not provide accurate biological results. Thus, in this proposed method initially, the static PPI data is converted into dynamic PPI data by integrating the gene expression data. Later, every dynamic subnetwork was clustered based on the popular clustering technique MCL along with the elephant herd optimization method for exploring and exploiting the better solution. The proposed method was employed on various 11 widespread datasets and the predicted complexes were compared with 4 different benchmark databases. Also, the proposed method was evaluated based on noise removal, insertion and deletion of random protein interactions, using the large PPI dataset, various parameter analyses, statistical significance and biological significance. On every evaluation phase, the proposed method was outperforming all other existing approaches and identified the common protein complexes, Gene Ontology functions and KEGG pathways of predicted protein complexes. As a future work, additional information on the unknown protein complexes predicted by the proposed method is to be addressed with the help of biological experts. The proposed method can also be applied and analyzed on weighted PPI networks. Also, various other diseased databases can be used to experiment.

## Supplementary information

Supplementary Information

## References

[1] Yang, C., Ji, J. & Lv, J. Identifying Protein Complexes Method Based on Time-sequenced Association and Ant Colony Clustering in Dynamic PPI networks. *Proc. IEEE 16th Int Conf on Bioinfo and Bioeng*, 21–27 (2016).

[2] Bader, G. D. & Hogue, C. W. V. An automated method for finding molecular complexes in large protein interaction networks. *BMC Bioinfo*, **4**(2) (2003). 10.1186/1471-2105-4-2 PMC149346 12525261

[3] Adamcsek B, Palla G, Farkas IJ, Derényi I, Vicsek T (2006). CFinder: locating cliques and overlapping modules in biological networks. Bioinformatics.

[4] Dongen, V. *Graph clustering by flow simulation*. (Ph.D. thesis, University of Utrecht, 2000).

[5] Wu, M., Li, X., Kwoh, C.K. & Ng, S.K. A core-attachment based method to detect protein complexes in PPI networks. *BMC Bioinformatics* **10**(169) (2009). 10.1186/1471-2105-10-169 PMC2701950 19486541

[6] Nepusz T, Yu H, Paccanaro A (2012). Detecting overlapping protein complexes in protein-protein interaction networks. Nat Methods..

[7] King AD, Przulj N, Jurisica I (2004). Protein complex prediction via cost-based clustering. Bioinform..

[8] Liu G, Wong L, Chua H (2009). Complex discovery from weighted PPI networks. Bioinformatics..

[9] Maulik, U., Basu, S. & Ray, S. Identifying protein complexes in PPI network using non-cooperative sequential game. *Sci Rep*, **7**(8410), (2017). 10.1038/s41598-017-08760-x PMC5566343 28827597

[10] Seckiner SU, Eroglu Y, Emrullah M, Dereli T (2013). Ant colony optimization for continuous functions by using novel pheromone updating. Appl. Math. Comput..

[11] Zhang Yijia, Lin Hongfei, Yang Zhihao, Wang Jian, Li Yanpeng, Xu Bo (2013). Protein Complex Prediction in Large Ontology Attributed Protein-Protein Interaction Networks. IEEE/ACM Transactions on Computational Biology and Bioinformatics.

[12] Lakizadeh, A. & Jalili, S. BiCAMWI: A Genetic-Based Biclustering Algorithm for Detecting Dynamic Protein Complexes, *PLoS ONE* **11**(7) (2016). 10.1371/journal.pone.0159923 PMC4963120 27462706

[13] Shih YK, Parthasarathy S (2012). Identifying functional modules in interaction networks through overlapping Markov clustering. Bioinform..

[14] Kennedy, J. & Eberhart, R. C., Particle swarm optimization, Proc of IEEE Int Conf on Neural Networks, IV, Piscataway, NJ, IEEEPress, 1942–1948. (1995).

[15] Ma, Q. & Lei, X. Application of artificial fish school algorithm in UCAV path planning. *Proc IEEE Fifth Int Conf on BioIns Comp: Theoand Appl*, 555–559. (2010).

[16] Lei X, Wang F, Wu FX, Zhang A, Pedrycz W (2016). Protein complex identification through Markov clustering with firefly algorithm on dynamic protein–protein interaction networks. Info Sci.

[17] Wang J, Peng X, Li M, Pan Y (2013). Construction and application of dynamic protein interaction network based on time course gene expression data. Proteomics.

[18] Vlasblom, J. & Wodak, S. J. Markov clustering versus affinity propagation for the partitioning of protein interaction graphs. *BMC Bioinform*. **10**(99) (2009). 10.1186/1471-2105-10-99 PMC2682798 19331680

[19] Wang GG, Deb S, Gao XZ, Coelho LDS (2016). A new metaheuristic optimisation algorithm motivated by elephant herding behaviour. Int Jnl of Bio-Ins Compu.

[20] Tuba V, Beko M, Tuba M (2017). Performance of Elephant Herding Optimization Algorithm on CEC 2013 real parameter single objective optimization. WSEAS Trans on Sys.

[21] Xenarios I (2002). DIP, the Database of Interacting Proteins: A Research Tool for Studying Cellular Networks of Protein Interactions. Nuc Acids Res.

[22] Stark C (2006). BioGRID: a general repository for interaction datasets. Nucleic Acids Res..

[23] Szklarczyk D (2017). The STRING database in 2017: quality-controlled protein-protein association networks, made broadly accessible. Nuc Acids Res..

[24] Gavin AC (2006). Proteome survey reveals modularity of the yeast cell machinery. Nature..

[25] Krogan NJ (2006). Global landscape of protein complexes in the yeast Saccharomyces cerevisiae. Nature..

[26] Collins SR (2007). Toward a comprehensive atlas of the physical interactome of Saccharomyces cerevisiae. Mol Cell Proteomics..

[27] Kiemer L, Costa S, Ueffing M, Cesareni G (2007). WI-PHI: a weighted yeast interactome enriched for direct physical interactions. Proteomics..

[28] Keshava Prasad TS (2009). Human Protein Reference Database–2009 update. Nucl Acids Res..

[29] Han K, Park B, Kim H, Hong J, Park J (2004). HPID: The Human Protein Interaction Database. Bioinfo..

[30] McDowall MD, Scott MS, Barton GJ (2009). PIPs: Human protein-protein interactions prediction database. Nucl Acids Res..

[31] Tu BP, Kudlicki A, Rowicka M, McKnight SL (2005). Logic of the yeast metabolic cycle: temporal compartmentalization of cellular processes. Science.

[32] Lapointe J (2004). Gene expression profiling identifies clinically relevant subtypes of prostate cancer. Proc Natl Acad Sci USA.

[33] Pu S, Wong J, Turner B, Cho E, Wodak SJ (2009). Up-to-date catalogues of yeast protein complexes. Nuc Acids Res..

[34] Mewes HW (2002). MIPS: a database for genomes and protein sequences. Nuc Acids Res..

[35] Cherry JM (1998). Saccharomyces Genome. Database: the genomics resource of budding yeast, Nuc Acids Res.

[36] Kikugawa, S. *et al*. PCDq: human protein complex database with quality index which summarizes different levels of evidences of protein complexes predicted from h-invitational protein-protein interactions integrative dataset. *BMC Syst Biol*, S2–S7 (2012). 10.1186/1752-0509-6-S2-S7 PMC3521179 23282181

[37] Aragues R, Garcia-Garcia J, Oliva B (2008). Integration and prediction of PPI using Multiple Resources from Public Databases. Jnl of Proteomics & Bioinfo..

[38] Lehne B, Schlitt T (2009). Protein-protein interaction databases: keeping up with growing interactomes. Hum Genomics..

[39] Li, X., Wu, M., Kwoh, C. K. & Ng, S. K. Computational approaches for detecting protein complexes from protein interaction networks: a survey. *BMC Genomics*. **11**(1) (2010). 10.1186/1471-2164-11-S1-S3 PMC2822531 20158874

[40] Kanehisa M, Furumichi M, Tanabe M, Sato Y, Morishima K (2017). KEGG: new perspectives on genomes, pathways, diseases and drugs. Nucleic Acids Res..

[41] Kanehisa M, Sato Y, Furumichi M, Morishima K, Tanabe M (2019). New approach for understanding genome variations in KEGG. Nucleic Acids Res..

[42] Kanehisa M, Goto S (2000). KEGG: Kyoto Encyclopedia of Genes and Genomes. Nucleic Acids Res..

