# Supplementary material for: Detection of dynamic protein complexes through Markov Clustering based on Elephant Herd Optimization Approach
Source: Sci Rep. 2019 Jul 31;9:11106. doi: 10.1038/s41598-019-47468-y (PMC6668483; doi:10.1038/s41598-019-47468-y)
Supplement: Supplementary file 1 — Supplementary Information [file 41598_2019_47468_MOESM1_ESM.docx]

**Supplementary Information**

**Detection of dynamic protein complexes through Markov Clustering based on Elephant Herd Optimization Approach**

**R.Ranjani Rani^1^, D.Ramyachitra^2*^, A. Brindhadevi^3^**

[ranjaniRSR91@gmail.com**^1^**](mailto:ranjaniRSR91@gmail.com1) **,** [jaichitra1@yahoo.co.in^2*^](mailto:jaichitra1@yahoo.co.in2*) , [brindharuna@gmail.com](mailto:brindharuna@gmail.com)^3^

**^1,2,3^ Department of Computer Science, Bharathiar University, Tamilnadu, India**

1. **Comparison of Various Accuracy Measures with various Datasets and Algorithms against MIPS Benchmark Dataset**


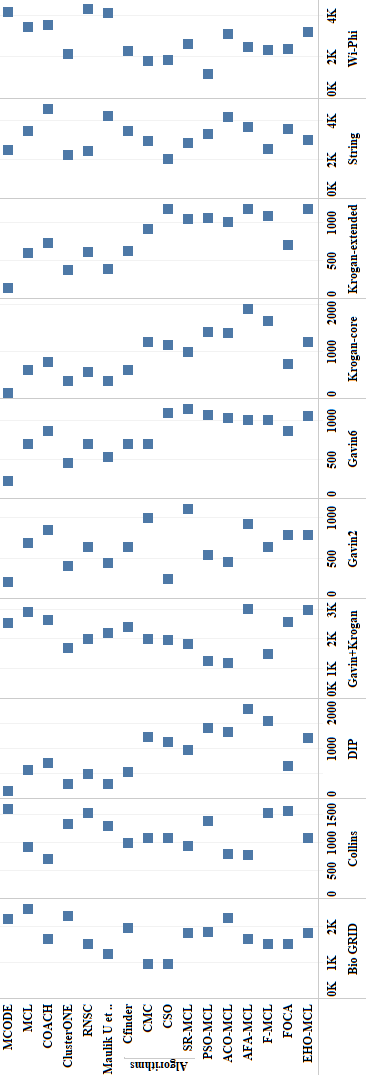


Fig S1 Comparison of Number of Clusters with various Datasets and Algorithms against MIPS Benchmark Dataset


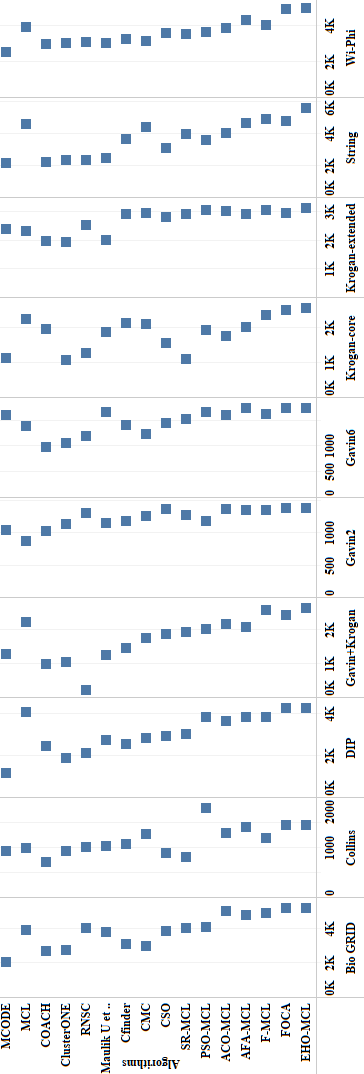


Fig S2 Comparison of Coverage Ratio with various Datasets and Algorithms against MIPS Benchmark Dataset


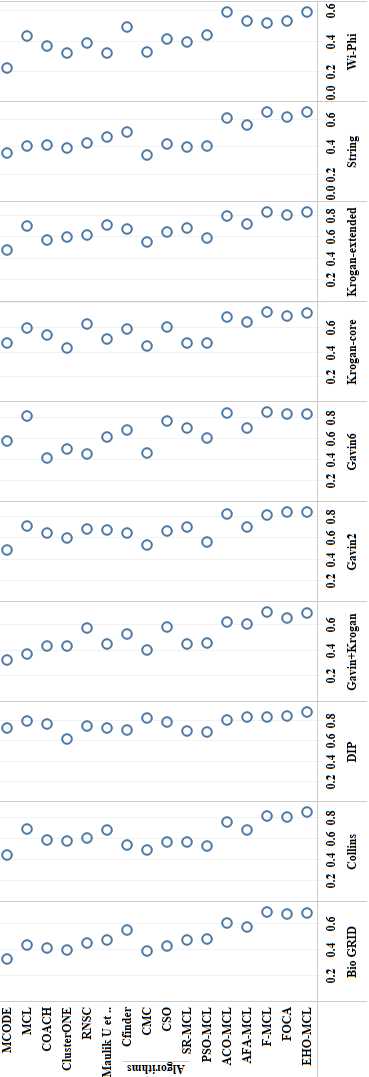


Fig S3 Comparison of Precision with various Datasets and Algorithms against MIPS Benchmark Dataset


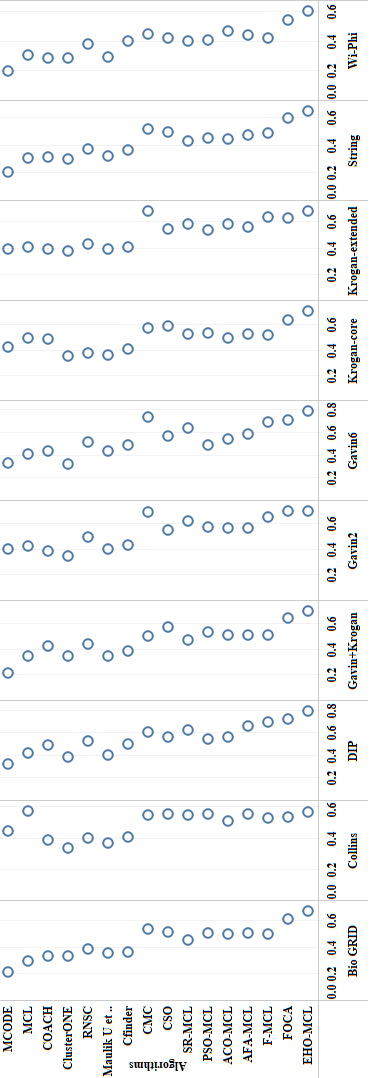


Fig S4 Comparison of Recall with various Datasets and Algorithms against MIPS Benchmark Dataset


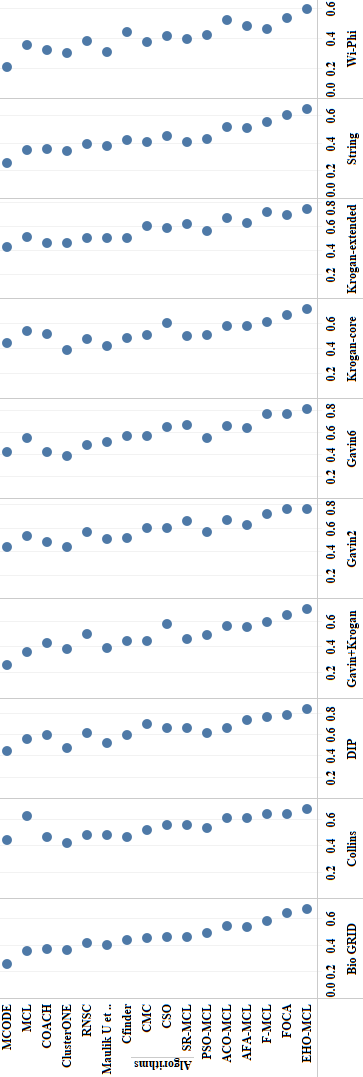


Fig S5 Comparison of F-Measure with various Datasets and Algorithms against MIPS Benchmark Datase


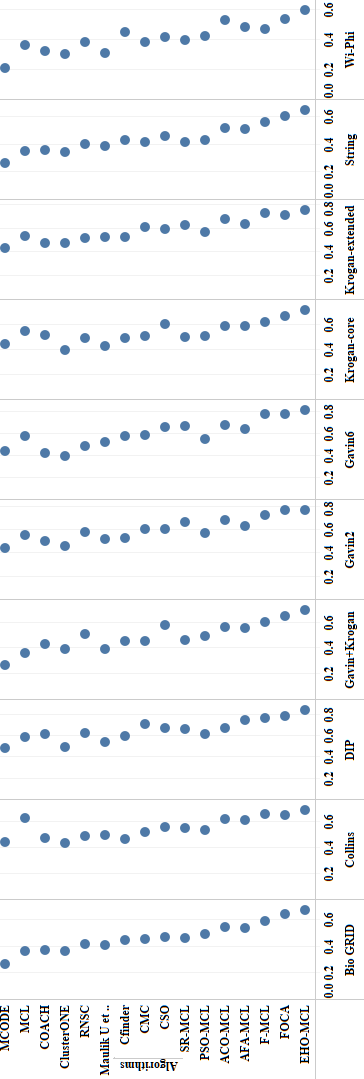


Fig S6 Comparison of Accuracy with various Datasets and Algorithms against MIPS Benchmark Dataset

1. **Comparison of Various Accuracy Measures with various Datasets and Algorithms against SGD Benchmark Dataset**


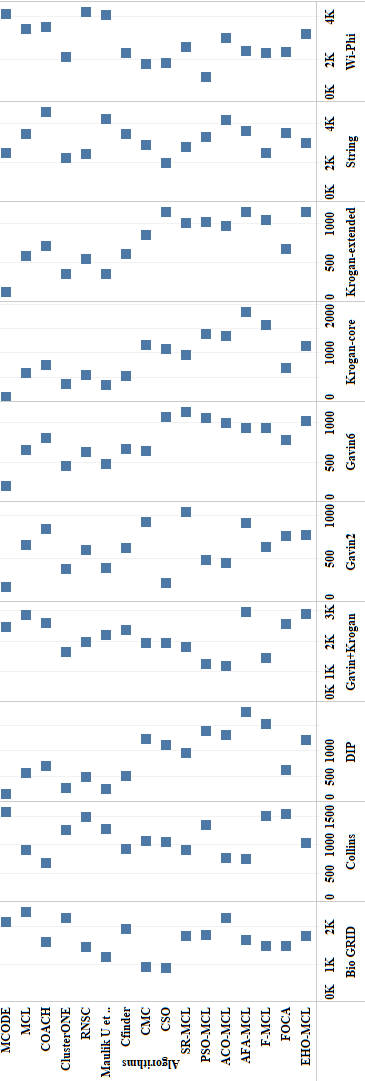


Fig S7 Comparison of Number of Clusters with various Datasets and Algorithms against SGD Benchmark Dataset


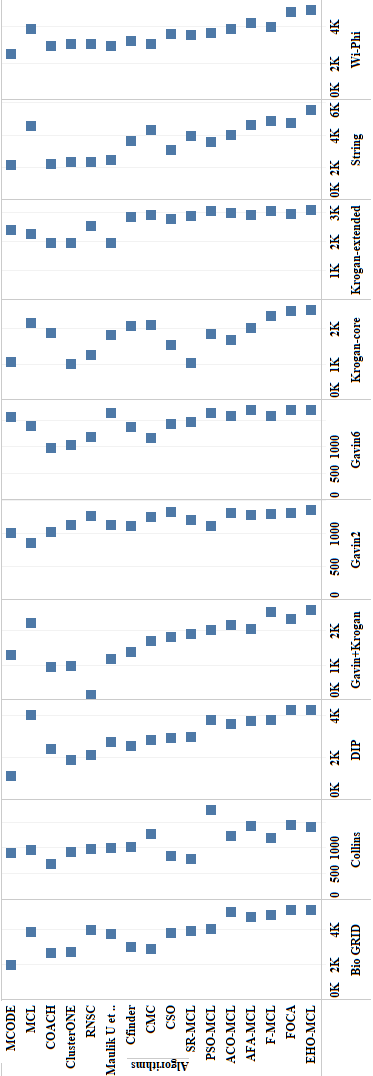


Fig S8 Comparison of Coverage Ratio with various Datasets and Algorithms against SGD Benchmark Dataset


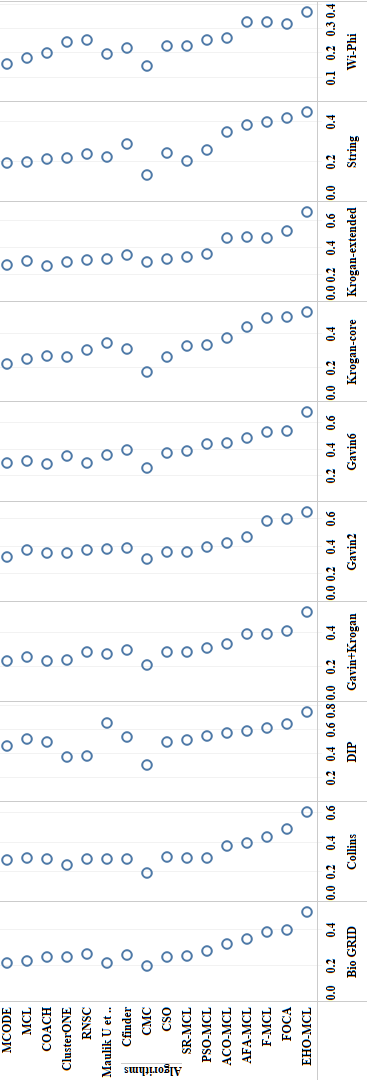


Fig S9 Comparison of Precision with various Datasets and Algorithms against SGD benchmark Database


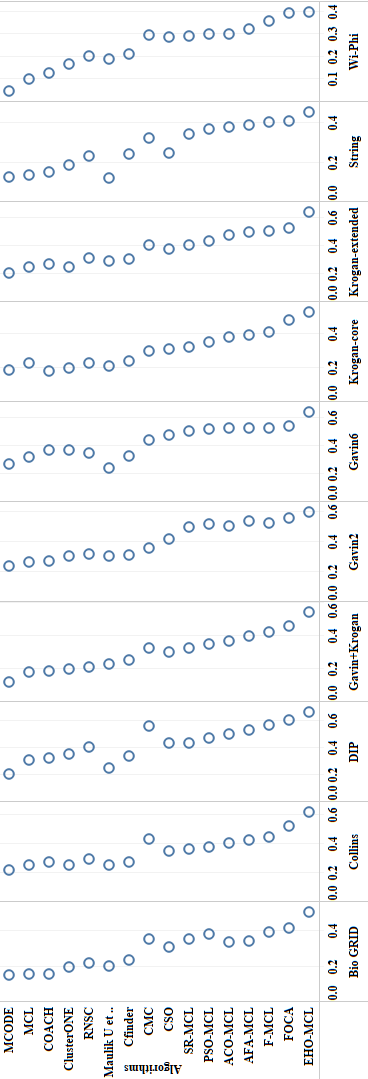


Fig S10 Comparison of Recall with various Datasets and Algorithms against SGD Benchmark Dataset


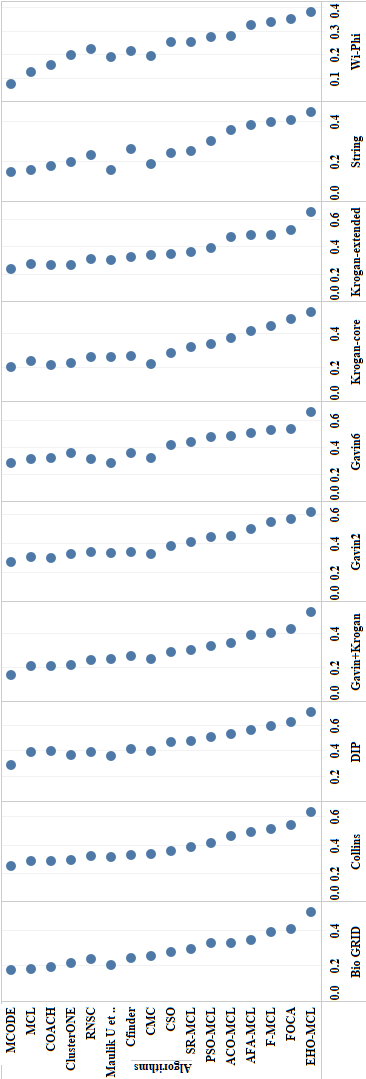


Fig S11 Comparison of F-Measure with various Datasets and Algorithms against SGD Benchmark Dataset


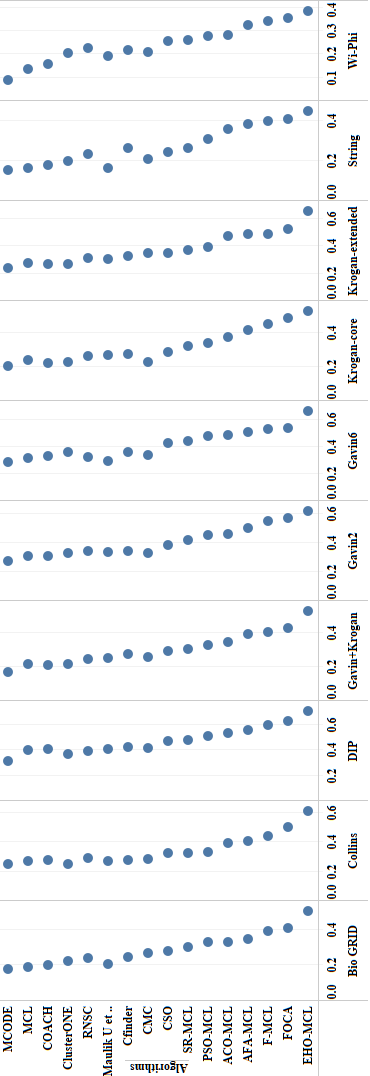


Fig S12 Comparison of Accuracy with various Datasets and Algorithms against SGD Benchmark Dataset

1. **Common KEGG Pathway of Predicted Protein Complex for Proposed Method.**

**
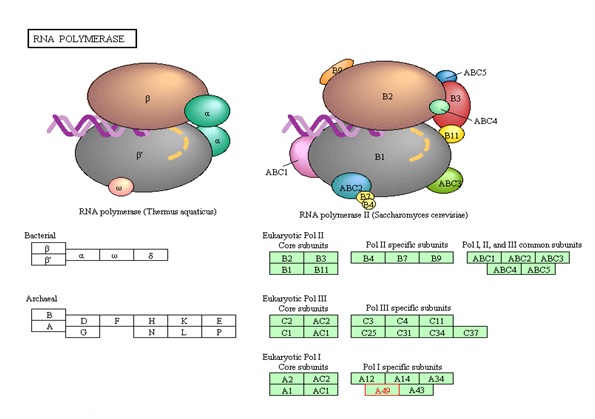
**

Fig S13 Diagram of common RNA Polymerase KEGG Pathway of predicted protein Complex by proposed method on Krogan-extended Dataset [1-3,5].

**
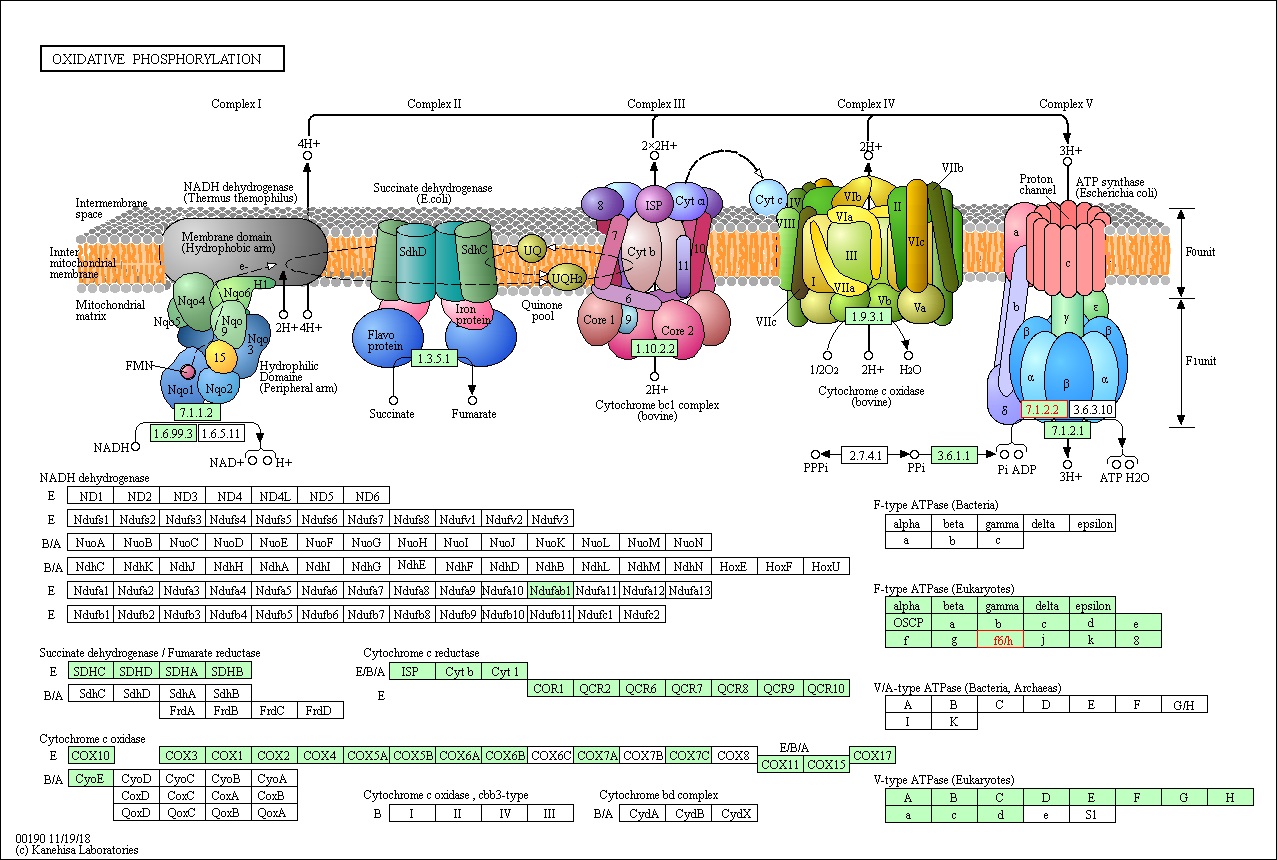
**

Fig S14 Diagram of common Oxidative Phosphorylation KEGG Pathway of predicted protein Complex by proposed method on DIP Dataset [1-4].

**References:**

1. Kanehisa, M., Furumichi, M., Tanabe, M., Sato, Y., & Morishima, K. KEGG: new perspectives on genomes, pathways, diseases and drugs. Nucleic Acids Res. 45, D353-D361, (2017).
2. Kanehisa, M., Sato, Y., Furumichi, M., Morishima, K., and Tanabe, M.; New approach for understanding genome variations in KEGG. Nucleic Acids Res. 47, D590-D595 (2019).
3. Kanehisa, M. and Goto, S.; KEGG: Kyoto Encyclopedia of Genes and Genomes. Nucleic Acids Res. 28, 27-30 (2000)
4. <https://www.kegg.jp/dbget-bin/www_bget?map00190>
5. <https://www.kegg.jp/dbget-bin/www_bget?map03020>
